# Relevance of temporal cores for epidemic spread in temporal networks

**DOI:** 10.1038/s41598-020-69464-3

**Published:** 2020-07-27

**Authors:** Martino Ciaperoni, Edoardo Galimberti, Francesco Bonchi, Ciro Cattuto, Francesco Gullo, Alain Barrat

**Affiliations:** 1 Aalto University, Espoo, Finland; 2 ISI Foundation, 10126 Turin, Italy; 3 University of Turin, Turin, Italy; 4 R&D Department, UniCredit, Milan, Italy; 5 CNRS, CPT, Turing Center for Living Systems, Aix Marseille Univ, Université de Toulon, 13288 Marseille, France; 6 Tokyo Tech World Research Hub Initiative (WRHI), Institute of Innovative Research, Tokyo Institute of Technology, Tokyo, Japan

**Keywords:** Complex networks, Statistical physics

## Abstract

Temporal networks are widely used to represent a vast diversity of systems, including in particular social interactions, and the spreading processes unfolding on top of them. The identification of structures playing important roles in such processes remains largely an open question, despite recent progresses in the case of static networks. Here, we consider as candidate structures the recently introduced concept of span-cores: the span-cores decompose a temporal network into subgraphs of controlled duration and increasing connectivity, generalizing the core-decomposition of static graphs. To assess the relevance of such structures, we explore the effectiveness of strategies aimed either at containing or maximizing the impact of a spread, based respectively on removing span-cores of high cohesiveness or duration to decrease the epidemic risk, or on seeding the process from such structures. The effectiveness of such strategies is assessed in a variety of empirical data sets and compared to baselines that use only static information on the centrality of nodes and static concepts of coreness, as well as to a baseline based on a temporal centrality measure. Our results show that the most stable and cohesive temporal cores play indeed an important role in epidemic processes on temporal networks, and that their nodes are likely to include influential spreaders.

## Introduction

A large variety of systems find a convenient representation as networks of interactions between components. Network representations have indeed proved to be useful to understand the structure and dynamics of systems as diverse as transportation infrastructures or social networks, as well as to describe processes occurring on top of them, such as information diffusion, epidemic spread, synchronization, etc^1^. A large body of work aims in particular at understanding how the network’s features impact the outcome of processes taking place on top of them, with the ambition of devising control and prediction capabilities. For instance, several methods have been put forward to identify the nodes of a network that play a more important role in a spreading process, either because they are “influencers” able to widely spread an information, or because they are “sentinels” with a high probability to be reached by a disease in its early stages and thus can give an early warning, or because their immunization is likely to hinder the spread^2–11^.

Such a task becomes even more complex when dealing with temporal networks, in which edges between nodes can appear and disappear on different time scales. The recent availability of time-resolved data sets of interactions has pushed network science beyond the static graph representation and has led to the development of the field of temporal networks^12,13^. The temporal dimension can yield non-trivial temporal features such as broad distributions of interaction times and of inter-event times (“burstiness”), heterogeneous activity distributions, causality constraints, and overall a broader diversity of activity/connectivity patterns than in static networks^12–15^. This has raised new questions on designing surveillance and control strategies for epidemic processes in temporal networks^16–23^. Identifying single nodes for the design of targeted interventions (immunization, isolation) can however be challenging in practice: indeed, centrality measures can fluctuate strongly over time and depend on details of the system that change in different periods; they might moreover be difficult to relate to interpretable features. Data with limited resolution can also make it difficult to correctly identify the most central nodes^24^. In particular, several works^17,18,21^ have addressed the issue of designing causal interventions, in which node properties are measured until a certain time and determine the nodes to immunize to contain a future spread. In practice, the impact of such strategies depends strongly on the data set under study and is limited by temporal fluctuations in the centralities of nodes. Moreover, action at the level of individuals is subjected to many informational and operational constraints.

Therefore, strategies based on intermediate scales have been advocated, targeting structures rather than single nodes, with the goal of identifying patterns that can be acted upon in practice. The idea in this case is to first identify potential structures of interest in (static or temporal) networks, and then verify that they play an important role in spreading processes: this is typically done by thought experiments in which one compares the outcome of a spreading process on the original network with the outcome if the structure of interest is altered or removed. Alternatively, one can seed the process from the structure and investigate the spreading power of nodes of the structure. A second important step is to relate the structures to interpretable features of the system of interest, and the third step is to use this knowledge to propose actionable strategies. Examples of such structures include groups or communities in the population, in which individuals have more contacts with each other than with the rest of the population, leading to the proposal of reactive measures of class closure^22,25^, or sets of links with correlated activity patterns^23^, which, in a school environment, can be related to the breaks between lectures and thus help design concrete interventions. We note here that the investigation of structures does not necessarily correspond to causal interventions in which only past knowledge is used. Rather, one wants to identify a certain type of structures that play an important role and could be interpreted in practical settings to be acted upon.

Here, we contribute to the first, most theoretical step of this line of research by proposing a novel type of candidate structures for idealized targeted interventions: the span-cores of a temporal network. The span-cores are structures that we recently introduced^26^ to decompose a temporal network into hierarchies of subgraphs of controlled duration and increasing connectivity, generalizing the core-decomposition of static graphs. They could thus in temporal contact networks be interpreted as long-lasting groups of interacting individuals. We recall that the core-decomposition of a static network yields a hierarchy of subgraphs that are increasingly densely connected: a core with coreness *k* is defined as the maximal subgraph such that all nodes within it have degree (number of neighbors in the core) at least *k*^27^. The nodes belonging to the more central cores have been shown to play an important role in standard theoretical models of epidemic spreading processes on static networks^4,28^.

In temporal networks, the span-cores are defined^26^ as temporal subgraphs as follows: a span-core ${{{\mathscr {C}}}}$ of order *k* is defined on an interval $\Delta$ of contiguous timestamps, such that all nodes in ${{{\mathscr {C}}}}$ have at all timestamps of that interval at least *k* stable neighbours in ${{{\mathscr {C}}}}$ (i.e., the links to these nodes are present during all timestamps of the interval). Each span-core is thus characterized by two quantities: its order and its duration (the length of the interval on which it is defined). Moreover, a span-core of order *k* on a temporal interval $[t_1,t_2]$ is said to be maximal if there exists no other span-core with order at least *k* and broader temporal interval ($[t'_1,t'_2]$ with $t'_1 \le t_1$ and $t'_2 \ge t_2$). In reference^26^, we have introduced the definition of span-cores and maximal span-cores, devised efficient algorithms to extract them from data, and performed a preliminary analysis of their properties in several data sets.

Maximal span-cores are thus well-connected stable groups of nodes, and can be considered as natural candidates when looking for structures having an important role in spreading processes on temporal networks. In the present work, we therefore study the role of the maximal span-cores of temporal networks in spreading processes occurring on these networks. To this aim, we perform two types of thought experiments. We first examine the impact of the removal of maximal span-cores with large order and/or duration on spreading processes in a variety of empirical temporal networks. We quantify this impact by the resulting decrease in epidemic risk and compare the removal of span-cores to random baselines and to the isolation of nodes based on properties in the static aggregated network known to be relevant to spreading processes^4,28^. For completeness, we also consider a strategy based on the computation of a centrality measure taking into account the whole temporality of the data, namely the temporal PageRank, which assigns a centrality measure to each node at each time^29^. We show that the removal of links in the most connected and stable temporal cores has a particularly strong impact on the epidemic risk. We then investigate the spreading influence of nodes belonging to maximal span-cores, defined as the final size of a spreading process originating in those nodes. Our results show that processes seeded in cohesive span-cores yield in general large epidemic sizes, even compared with several baseline seeding strategies based on properties known to be relevant such as static centralities. We note that we do not attempt to classify strategies based on all potential centrality measures: first, one can expect the classification to be highly dependent on specific data set properties; second, centrality measures identify nodes rather than structures. For our goal here, it is thus enough to check whether strategies based on span-cores yield reliably large effects. Overall, our results confirm the important role of the span-cores in diffusion processes on temporal networks. We discuss some limitations and perspectives in the “Discussion” section, in particular concerning the need to know the temporal network in advance to find the span-cores, showing that one has to be able to interpret the span-core structures in order to translate this theoretical knowledge into actionable strategies.

## Results

### Approach

Before presenting our results, we describe here our general methodology, and we refer to the “Methods” section for more details on each step of our procedure. We consider a series of publicly available data sets describing temporal networks of particular relevance for epidemic spreading scenarios, namely high-resolution social network data on face-to-face interactions in a variety of settings, collected independently by two different collaborations^30,31^: schools, conferences, offices, hospital (see “Methods” section for details). This allows us to study data with a broad variety of structural and temporal patterns. The data sets we consider are all represented as temporal networks on discrete timestamps: nodes represent individuals, and a temporal edge (*i*, *j*, *t*) between two nodes *i* and *j* in time window *t* corresponds to the fact that these two nodes have been in contact during that time window.

We consider standard schematic models of epidemic spreading processes occurring on top of these temporal networks, namely the Susceptible-Infected-Susceptible (SIS) and Susceptible-Infected-Recovered (SIR) models. In the SIS case, nodes can only be in either of two states: susceptible (S) or infected (I). A susceptible node in contact with an infected one becomes infected with a fixed probability $\beta$ per unit time. Infected nodes recover with a constant rate $\nu$ and become susceptible again. The system can thus either reach a steady state in which there is constantly a finite fraction of the nodes in the I state, or the epidemic can die out if all nodes recover. In the SIR model, nodes that recover enter the Recovered (R) class and can no longer take part in the epidemic process. Therefore, no steady state can be reached: the epidemic process ends when no more infected nodes are present, and all nodes are then either susceptible (those who have not been reached by the disease) or recovered.

To evaluate the outcome of these processes, we resort to two standard measures of the epidemic risk. We first take advantage of the theoretical framework developed by^32,33^ to compute the epidemic threshold for both SIS and SIR models in arbitrary temporal networks (see “Methods” section and^32,33^ for details). The epidemic threshold represents a crucial way of quantifying the epidemic risk in a system, as it gives the critical value of disease transmissibility above which the simulated pathogen is able to reach a large fraction of the population. In the SIR case, we moreover perform direct numerical simulations of the process and measure the epidemic size, i.e., the final fraction of recovered nodes, averaged over 1000 realizations of the process. Note that the process might not have ended at the end of the data set, meaning that there might still be nodes in the I state. We then replicate the temporal network until the process has ended. The epidemic size can be interpreted both as a further quantification of the epidemic risk and as the spreading power of the seed, i.e., the initial source of the spread.

As mentioned in the introduction, two complementary questions are typically investigated in order to test the role of nodes or structures in spreading processes: how to best contain or mitigate a spread, and which seed(s) have the largest spreading power? Containment and mitigation aim at increasing the epidemic threshold and/or at decreasing the final epidemic size, and various strategies can be considered and compared. Here we focus on the role of the maximal span-cores, and thus consider an idealized strategy based on removing from the temporal network a fraction of these structures. We thus first determine in each data set its maximal span-cores (see “Methods” section for the precise definitions). We then consider an altered version of the temporal network in which a fraction *f* of the temporal links, taken from the maximal span-cores, are removed: for a span-core spanning a temporal interval $[t_1,t_2]$ this amounts to removing the temporal links in which any node of the span-core is involved between $t_1$ and $t_2$ (i.e., effectively isolating these nodes during $[t_1,t_2]$). The span-cores on which this strategy is applied are chosen either as the ones with the largest order *k* (*k*M maximal span-cores strategy), or the ones with the largest duration $\Delta$ ($\Delta$M maximal span-cores strategy): we remove temporal edges starting from the ones of the maximal span-cores ordered by non-increasing order or duration, until the fraction *f* is reached. This corresponds to removing all the temporal edges of a certain number of maximal span-cores, that we denote $n_{msc}$, and potentially some additional temporal edges taken at random in the next maximal span-core (number $n_{msc}+1$ in the ordered list) to reach exactly *f*. For a given data set, the total number of temporal edges removed is denoted $n_T$ ($n_T = f |E|$ where *E* is the overall set of temporal edges in the data set). Moreover, for a given strategy *s*, we denote by $n_t^s$ the number of temporal edges removed at timestep *t* in this strategy. Finally, we compare the epidemic risk in the altered temporal network and in the original one: specifically, we measure the relative variation of the epidemic threshold with respect to its value in the original temporal network, and we compare (for the SIR case) the average final epidemic size in both cases.

Moreover, and to assess the scale of the impact of the removal of maximal span-cores, we compare the results to several alternative strategies for removing temporal edges, and in particular to some strategies based on properties known to be important for spreading processes. To perform a sensible comparison, each of these alternative strategies must consist in the removal of a globally equivalent number of temporal edges $n_T$, to have overall the same impact on the global temporal network activity. We first consider strategies (see details in “Methods” section) based on static measures of coreness, since static cores have been shown to play a role in spreading processes: these would be effective strategies if the processes were taking place on temporally aggregated networks. To this aim, we aggregate the temporal network on the data set temporal window: in the aggregated network, two nodes are connected if they are connected at least once in the temporal network, and the corresponding static edge has a weight equal to the number of timestamps with a temporal edge between them. We perform the static *k*-core decomposition as well as its weighted counterpart *s*-core decomposition^34^ and then consider sequentially the nodes starting from the cores with highest order (either *k* or *s*, yielding the SC and SWC strategies) and remove all the temporal edges of these nodes, until $n_T$ temporal edges have been removed (nodes are ordered at random in each core; also, whenever removing all temporal edges of a node would lead to a total number of removed edges larger than $n_T$, we remove edges of this node at random until we reach exactly $n_T$). Although our focus is on structures rather than single nodes, we also consider strategies based on the most well-known node centrality measures in the aggregated network (degree and strength, yielding the SD and SWD strategies), proceeding in the same manner. As several centrality measures have been generalized to temporal networks, we moreover consider a strategy based on computing the temporal PageRank^29^ of each node at each time, classifying the pairs (*node*, *time*) according to this centrality and removing temporal edges of nodes using this classification (tPR strategy). Finally, we consider three random baselines strategy. In the “random times” (RT) strategy, $n_T$ temporal edges are removed totally at random from the temporal network. In the “random by timestamp”, we remove exactly $n_t^s$ temporal edges at random at each timestep *t*: there are thus two such strategies, kRTT (resp. $\Delta$RTT) removing the same number of edges at each time step than the *k*M (resp. $\Delta$M) maximal span-cores strategy. This means that these strategies are informed by the temporality of the maximal span-cores, but not by which nodes and edges they contain.

To investigate on the other hand whether nodes belonging to maximal span-cores tend to have a high spreading power, we proceed as follows: we compute the ratio of the average epidemic sizes obtained (1) when a node of a maximal span-core is chosen as seed of the SIR process and (2) when a random node is chosen instead (see “Methods” section). We then compare the epidemic ratio obtained for nodes chosen in maximal span-cores and for nodes chosen according to aggregated static characteristics (nodes of the static cores with highest order, nodes with high degree, nodes with high strength).

While we perform these investigations for 8 different data sets (see “Methods” section), we show in the main text the results for two data sets corresponding to: a high school (where the aggregated contact network displays a clear community structure, and contacts between classes are observed only during the breaks, giving rise to interesting correlations between structure and temporal patterns^23,35^) and a workplace (where group structure is much weaker and individuals mix with no time constraints^36^). The results for the other data sets are shown in the Supplementary Material.

**Figure 1:**
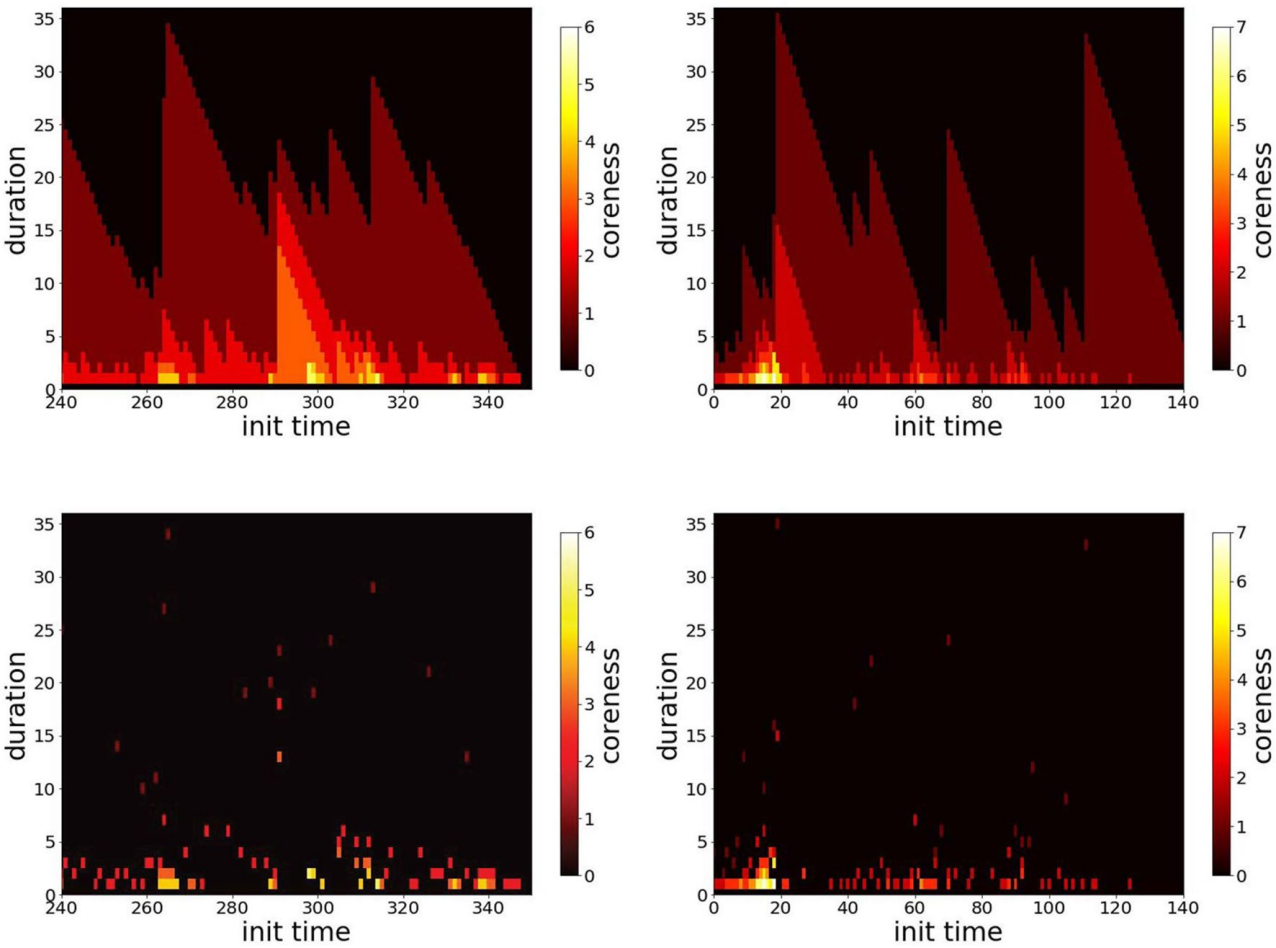
Colorplot of the span-cores temporal activity for two data sets. Left column: High School. Right column: Workplace. Top plots: all span-cores. Bottom plots: maximal cores. We restrict in each case to 1 day of data for a better visibility. In each plot, the x axis reports the timestamp at which the span of a span-core starts, the y axis specifies the size of the span (in minutes), and the color scale shows its order (coreness) k.

**Figure 2:**
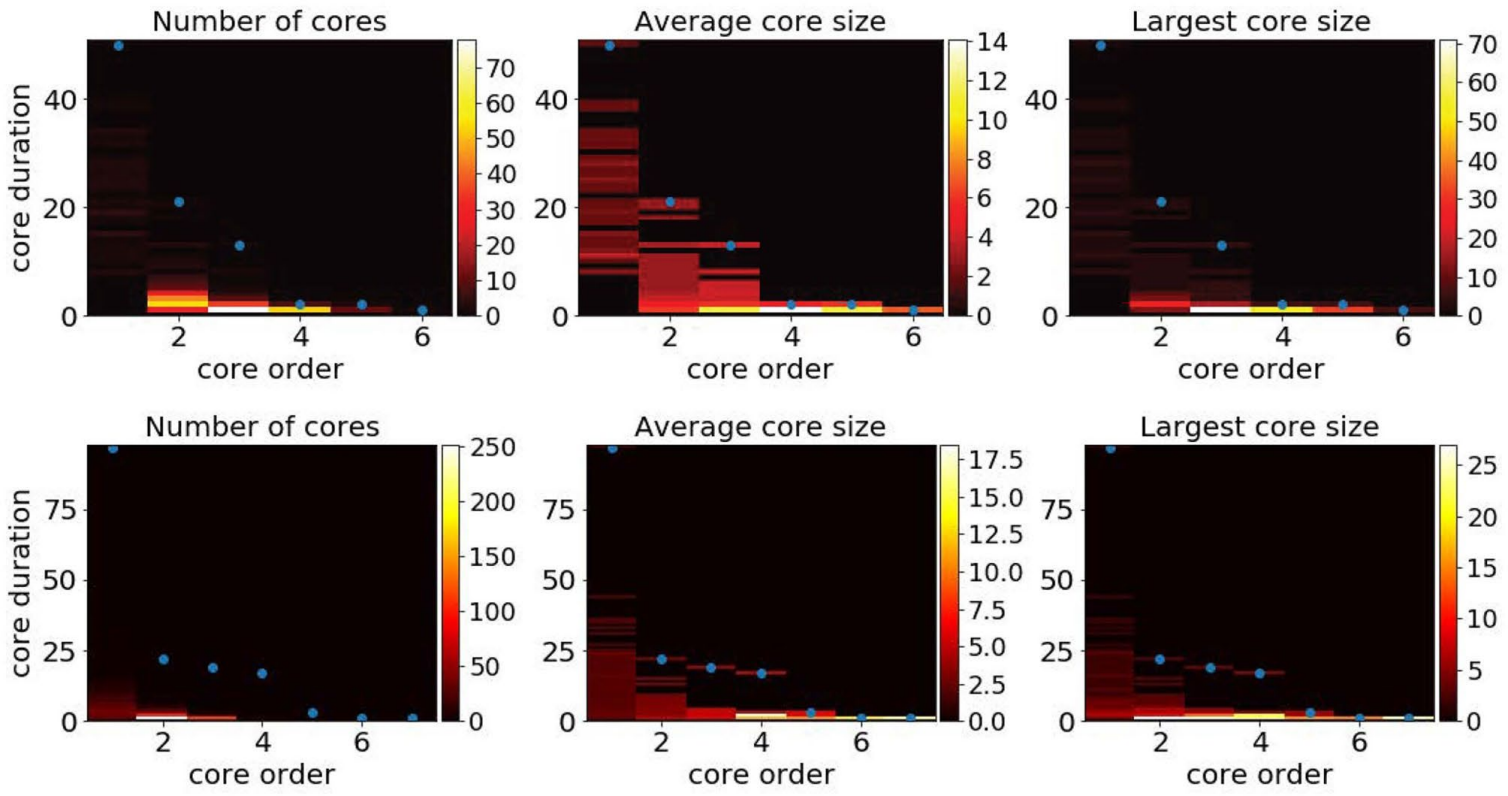
Aggregated statistics of the maximal span-cores for two data sets. Top row: High School. Bottom row: Workplace. Each panel shows with a color scale the property of cores with given core order (x-axis) and core duration (y-axis). Left plots: number of cores. Middle plots: average size (number of nodes) of these cores. Right plots: size (number of nodes) of the largest of these cores. The blue dots give, for each order, the maximal duration of cores with that order.

### (Maximal) span-core statistics

Let us first discuss some statistics regarding the maximal span-cores found in the temporal networks, which are here the structures of interest targeted by the intervention strategies. Figure 1 presents the timeline of the span-cores of the High School and Workplace data sets. As each span-core is characterized by a temporal interval (its span) and an order, we resort to a colorplot for their visualization: in each plot, the x-axis corresponds to the starting time of the interval, the y-axis to the duration of the core, and the color encodes its order. This allows to highlight periods in which cores with long durations and/or high order are observed. Note that the triangular shapes observed in the timelines showing all span-cores are a direct consequence of the span-cores definition: if a span-core of order *k* is present on an interval $\Delta = [t_1,t_2]$, then there is also a span-core of the same order on all the intervals $[t_1+1,t_2],\cdots ,[t_2-1,t_2], [t_2,t_2]$, with respective durations $|\Delta |-1$, ..., 2, 1. Note also that it was shown in Ref^26^, that the observed timeline is not trivially linked to the activity timeline: reshuffled data sets in which the number of temporal edges in each timestamp is conserved, as well as the instantaneous degree of each node, yield indeed span-cores with durations and orders systematically much lower than in the original data.

Figure 2 and Table S1 in the Supplementary Material present some aggregated statistics of the maximal span-cores, aggregated over the whole temporal network (see also “Methods” section). We show in particular, as a function of core order and duration, the number of maximal span-cores, their average number of nodes and the largest number of nodes of a maximal span-core with these order and duration values. We also highlight the largest core duration found at a given core order: for larger values of the order, the largest duration decreases, and the largest order cores have duration 1, corresponding to cohesive groups that last only briefly. On the other hand, some cores of order 1 can last for many timestamps: they correspond to the most stable contacts between two nodes.

While these statistical properties do not provide a detailed temporal information as in Fig. 1, they nonetheless give an idea of the richness of the temporal patterns present in a data set. To highlight this point, we show in the Supplementary Material how the results of Fig.  2 are changed when the data is temporally reshuffled using several null models: as there are many possible null models for temporal networks^37^, we consider several reshuffling possibilities, which all preserve the global activity timeline. In all cases, the reshuffled data show a clearly less rich core structure, with in particular smaller maximal order at a given core duration.

Finally, we mention that span-cores might be used to give a time-dependent measure of centrality of each node in the temporal network: one can for instance at each timestamp take into account the span-cores to which a node belongs, and consider as centrality measure the highest order of these cores. One can also measure the duration of these cores, their size, etc. One can then aggregate these measures over the whole temporal window, to obtain the maximal core order to which a node belongs, or the average order, etc, yielding global centrality measures for each node based on the temporal structures to which it contributes. In the Supplementary Material we show that these measures are statistically correlated with static coreness measures in the static network obtained by temporal aggregation of the temporal network, but that the correlation is weak and many outliers are observed: this is expected as a given node’s static connectivity properties in the aggregated network might come from the results of non-simultaneous interactions, while span-cores are defined by cohesiveness properties that are local in time.

### Impact of removal strategies on activity timelines

As mentioned above and detailed in the “Methods” section, in order to assess the relevance of these structures, we consider as idealized intervention strategies to mitigate spreading processes the removal of maximal span-cores, as well as other strategies based on static centrality measures that are known to be relevant for spreading processes^2,4^.

**Figure 3:**
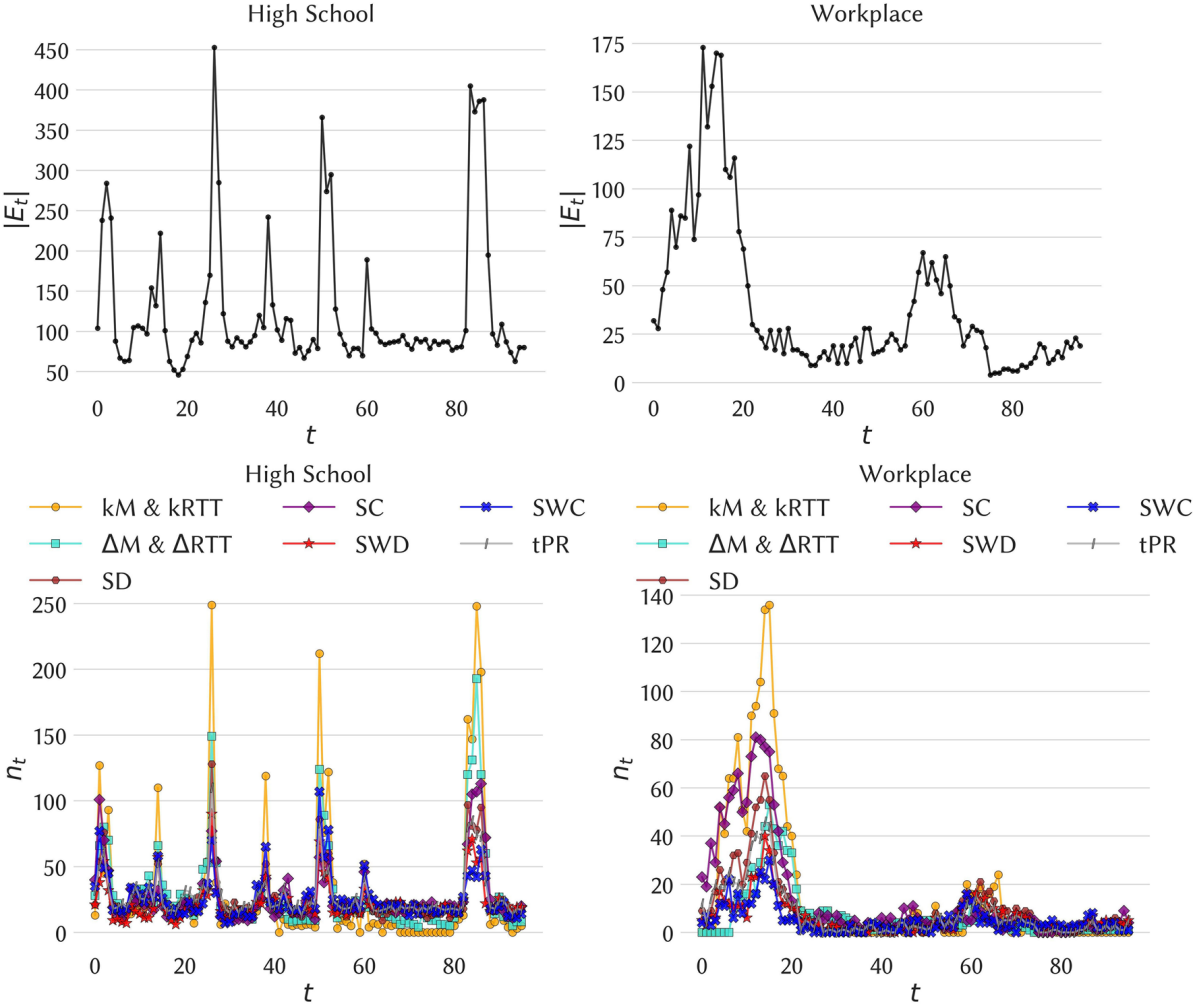
Original activity timelines (number of temporal edges per timestamp, top plots) and number of temporal edges removed per timestamp for each strategy (bottom plots), for 1 day of data. Here the overall fraction of removed temporal edges is $f=20\%$. The kM and $\Delta$M strategies target maximal span-cores (respectively with largest order and largest duration); the kRTT and $\Delta$RTT remove the same number of temporal edges at each time as the kM and $\Delta$M strategies, respectively, but choosing the edges at random; the SD, SWD, SC and SWC are based on static quantities measured on the aggregated network, namely degree, strength, coreness and weighted coreness, respectively (See “Methods” section for the detailed list of strategies). Left column: High School. Right column: Workplace. The values of the total numbers of removed span-cores and temporal edges are given in Table 1.

Table 1 gives, for each strategy based on span-cores, some properties of the span-cores targeted. As we consider the overall removal of a fixed fraction of the temporal edges (here $f=20\%$), the number of span-cores targeted depends on the strategy. Moreover, targeting the cores with largest order (kM strategy) leads to the removal of cores with smaller duration but larger size than the $\Delta$M strategy. Figure 3 shows the impact on the activity timelines of the various strategies: each curve gives the number of temporal edges removed at each time step (i.e., $n_t$ vs *t*) for a specific strategy. The figure shows that all strategies tend to remove more temporal edges in periods of high activity. This effect is however much stronger for the strategies based on span-cores, especially for the kM strategy: this is linked to the fact that large order cores appear during these periods. On the other hand, strategies based on static measures or on temporal PageRank remove also a substantial number of temporal edges during low-activity periods. Note that the $\Delta$M strategy strikes a balance, in particular in the high school case, removing more temporal edges than the static strategies in the large activity periods but also more than the kM strategy in low activity periods (this is due to the fact that some cores with large duration have order 1, corresponding for instance to long-lasting links that extend beyond large activity periods, see Figs. 1 and 2).

**Table 1:** Basic properties of the maximal span-cores removed in each of the targeted strategies (with removal of $f=20\%$ of the temporal edges). $n_{msc}$: number of maximal span-cores with all temporal edges removed; $\langle k \rangle$: average order of the targeted maximal span-cores; $\langle |\Delta | \rangle$: average duration of the targeted maximal span-cores; $\langle n \rangle$: average number of nodes in the targeted maximal span-cores; $\langle e \rangle$: average number of temporal edges removed per time step impacted by the strategy.

Data set	Strategy	$n_{msc}$	$\langle k \rangle$	$\langle |\Delta | \rangle$	$\langle n \rangle$	$\langle e \rangle$
High school	Largest order cores	300	2.98	2.72	7.97	40.56
	Largest duration cores	289	2.13	7.17	4	43.24
Workplace	Largest order cores	232	2.74	2.46	5.20	27.46
	Largest duration cores	179	1.13	10.50	2.16	30.71

### Impact of removal strategies on the epidemic risk

**Figure 4:**
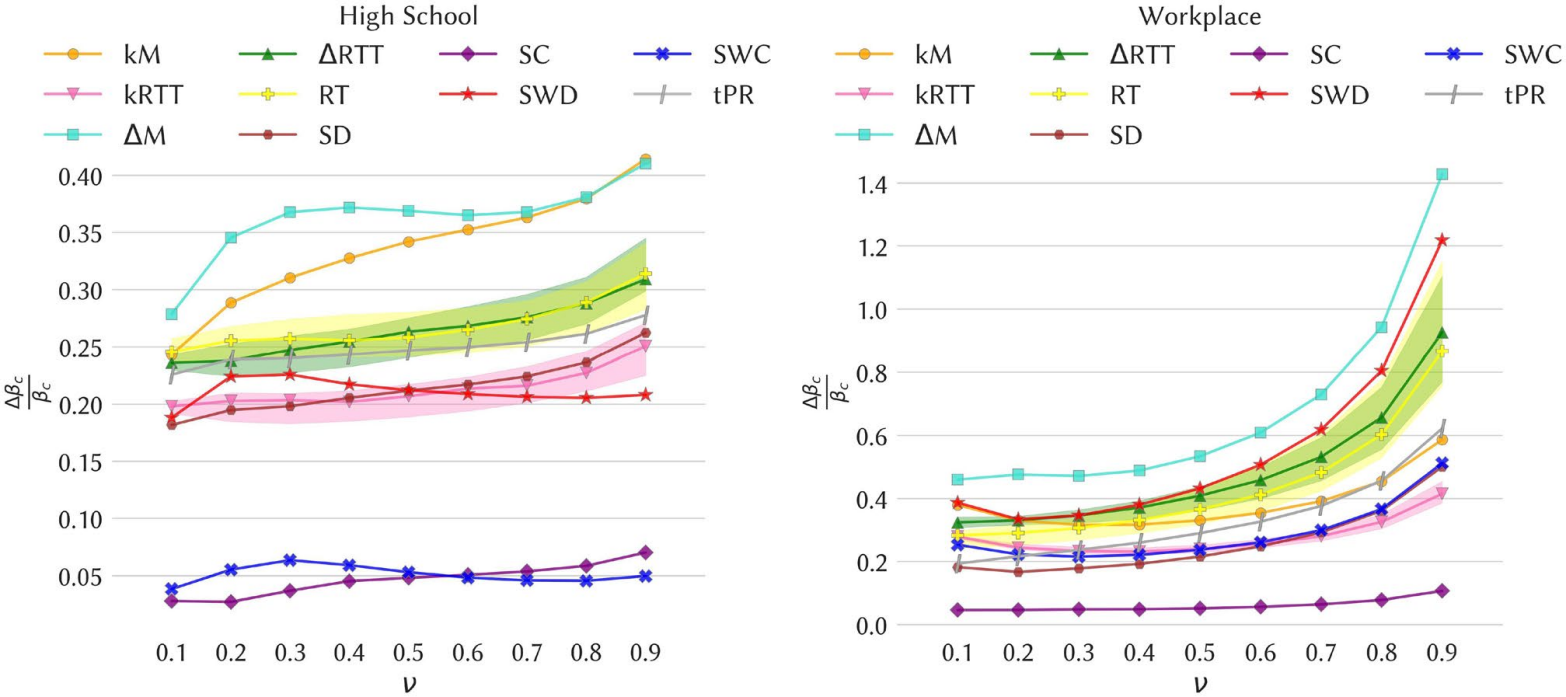
Impact of the various intervention strategies as measured by the change in the epidemic threshold of SIS processes. Left plot: High school. Right plot: Workplace. In each panel we plot for the various strategies the relative change $\Delta \beta _c / \beta _c$ in the epidemic threshold of an SIS process on the temporal network, computed using the method of Valdano et al.^32^, as a function of the recovery rate $\nu$. For each strategy based on random choices, we show the confidence interval between the $5{\mathrm{th}}$ and $95{\mathrm{th}}$ percentiles as a shaded area (computed on 30 samples).

Figure 4 shows the impact of the various removal strategies on the epidemic threshold of SIS and SIR spreading processes for the data sets High School and Workplace (results for the other data sets are shown in the Supplementary Material). The figure shows, as a function of the recovery rate $\nu$, the relative variation $\Delta \beta _c / \beta _c$ between the threshold computed on the temporal network after removal of temporal edges and the threshold in the original temporal network. In all cases the variation is positive, meaning as expected that the removal of edges increases the epidemic threshold and thus decreases the epidemic risk.

**Figure 5:**
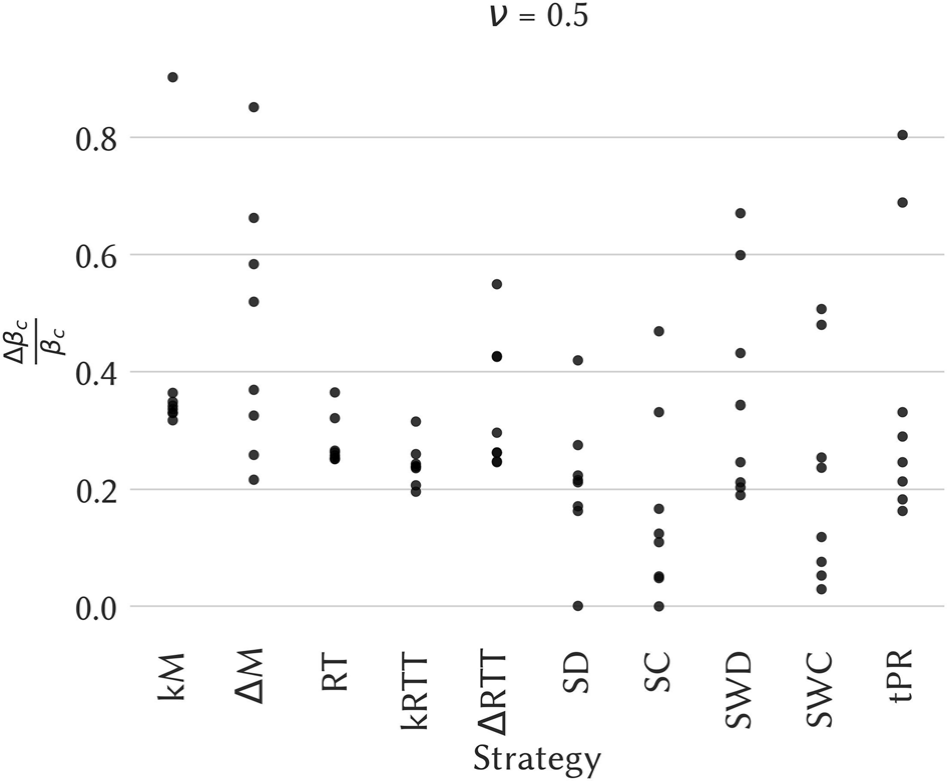
Epidemic threshold relative variation $\Delta \beta _c / \beta _c$ for $\nu = 0.5$ over all the 8 datasets and candidate strategies. For a given strategy, indicated on the *x*-axis, each point corresponds to one data set and its *y*-axis value is given by $\Delta \beta _c / \beta _c$.

We first observe that the size of the effect varies a lot for different strategies and also between data sets: some strategies lead only to a small shift of the epidemic threshold, while in some cases its value can be doubled. We also note that the best strategy, and more generally the classification of strategies according to the size of the epidemic threshold shift, depends on the data set under consideration. However, several points are worth highlighting. First, the best strategy is almost always the $\Delta$M or the kM one, i.e., strategies based on maximal span-cores. Moreover, in school contexts the kM strategy is either best or second-best, and in three cases out of four the two span-cores based strategies are the best performing ones. Finally, even when they are not the two best ones, the strategies based on maximal span-cores always have a large impact on the epidemic threshold, while the effect of strategies based on static centrality measures is more variable across data sets. This is illustrated in Fig. 5 that shows, for a specific value of $\nu$, the eight values of $\Delta \beta _c / \beta _c$ for each strategy (one value per data set). The results are consistently large for the span-core strategies. Strategies based instead on static coreness (either weighted or not) and on static degree have in some cases a very small impact, while the static strength strategy, that targets nodes according to their total number of contacts, also yields a strong impact for all data sets, as well as the tPR strategy that in addition integrates temporal information. We however note that both these strategies target single nodes (and the tPR strategy even isolates single nodes at specific times) and not whole structures as the span-core strategies do and hence carry an advantage in the comparison, despite being less actionable in practice. We moreover show in the Supplementary Material that these results are robust with respect to a change in the fraction of removed temporal edges.

As a further investigation, we have considered the epidemic size reached by the SIR process on temporal networks altered by the various intervention strategies. While all strategies tend to decrease the epidemic size, we show in the Supplementary Material that there is no clear optimal strategy: which strategy performs best depends both on the spreading parameters and on the data set, and in many cases the epidemic size mitigation is of similar amplitude for the various strategies. In particular, the removal of span-cores has thus an impact as important as other well-known strategies using single node centralities.

### Impact of seeding strategies on the epidemic sizes of SIR processes

**Figure 6:**
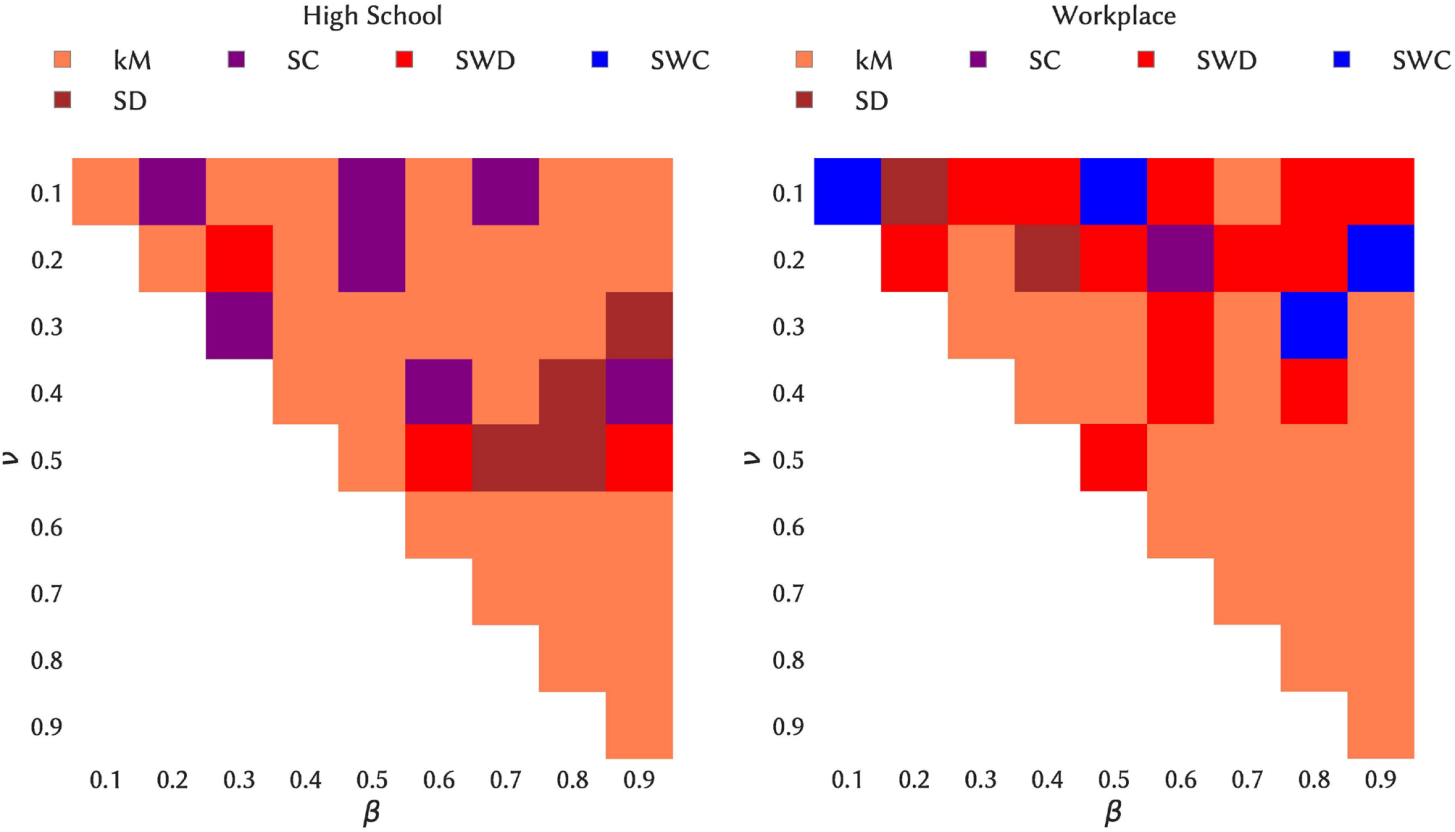
Heatmap indicating the seeding strategy leading to the largest ratio of average final sizes, for each combination of spreading parameter values. Left column: High School. Right column: Workplace.

**Figure 7:**
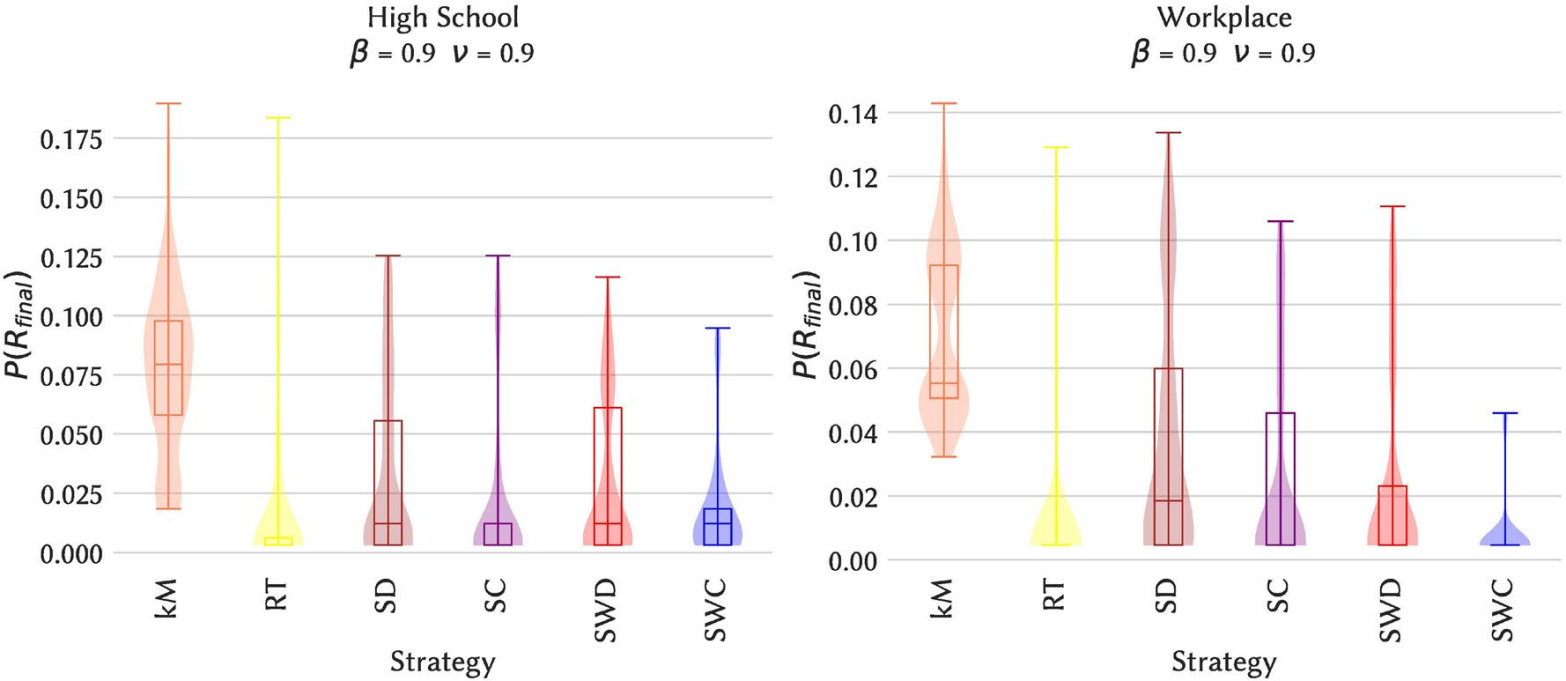
Distributions of final epidemic sizes for some illustrative cases, for an SIR process on the original temporal network and for several seeding strategies. The RT case corresponds to the baseline of randomly chosen initial time and seed. Left column: High School. Right column: Workplace.

We now consider the issue of spreading maximization, namely, which choice of the initial seed and time of the spread leads to the largest spread. Here, we consider as possible seeding strategy an initial seed belonging to a span-core of high order, with an initial time of the spread at a time in the temporal interval of this span-core (kM strategy, see “Methods” section). Moreover, as nodes with large degree or strength and nodes belonging to cores of high order are known to have a large spreading power in static networks, we also consider processes originating in such nodes (see “Methods” section). Figure 6 shows, for the various combinations of spreading parameters, which strategy leads to the largest average epidemic size (see also Fig. 7 and the Supplementary Material for the other data sets). While some dependency on the parameter values is observed, seeds chosen in maximal span-cores of high order have consistently a large spreading power, and in fact in most cases the largest among the considered baselines. Moreover, we show in the Supplementary Material that the spreading power tends to increase with the order of the span-core to which the seed belongs.

## Discussion

In this work, we have investigated the relevance of specific temporal structures, namely the span-cores put forward in Ref.^26^, for spreading processes on temporal networks. The span-core decomposition generalizes to temporal networks the *k*-core decomposition of static graphs, by extracting in each time interval subgraphs of increasing internal connectivity (“order”). Given their definition as lasting well-connected structures, it is rather intuitive that span-cores of high order and/or duration might play an important role in propagation processes on temporal networks, just as static cores of large coreness are relevant for such processes on static networks^4,28^. We therefore focused here on the maximal span-cores: a span-core on an interval $\Delta$ is defined as maximal if no other span-core can be found with higher order and on a broader temporal interval than $\Delta$.

To test the relevance of the maximal span-cores on propagation processes, we have investigated how two standard simplified models for epidemic spreading processes, the SIS and SIR models, are impacted by the removal of a fraction of the temporal edges forming these span-cores, starting with the cores of highest order or of longest duration: this corresponds to temporary “quarantining” the nodes involved in these cohesive structures. We have compared the impact of such mitigation strategies to several baselines strategies of temporal edge removal targeting nodes chosen according to their centrality (degree or coreness, both weighted or not) in the static network obtained by aggregation of the temporal network, as these properties are known to impact spreading processes. We have also considered a strategy based on the generalization of PageRank to temporal networks, which allows to define a centrality that is local in time for each node.

We have quantified the impact of these strategies on the one hand by measuring the induced relative shift in the epidemic threshold of the process, and on the other hand, in the SIR case, by the change in the resulting epidemic size (i.e., the fraction of the population affected by the spread). We have performed these numerical experiments using several empirical temporal networks of relevance for epidemic spread, namely data describing human interactions in a variety of contexts.

The results show that the shift in epidemic threshold resulting from the removal of temporal edges in maximal span-cores is consistently large; the strategies of removing edges in the span-cores of large orders or durations figure moreover among the mitigation strategies leading to the strongest impact among the considered strategies, while the impact of strategies based on static measures depends more on the specific data set. The good performance of the strategies based on maximal span-cores in school environments is particularly interesting as such contexts are of special relevance for containing infectious disease spread^22^ and span-cores might be related to interpretable events in such settings, such as groups forming during the breaks. The fact that strategies based on temporal structures perform well in such environment could be related to the fact that such environments present at the same time a strong community structure and also specific temporal patterns of correlated edges that cannot be exposed by purely static measures^14,15^. Overall, span-cores are thus more able to detect structures whose targeting leads to an increase of the epidemic threshold than static quantities. In terms of final epidemic size, we note that all strategies considered lead to a large decrease of this epidemic risk measure of comparable size, confirming the consistently important role of span-cores.

We have also investigated the spreading power of nodes belonging to span-cores of high order, comparing the final size of SIR processes seeded at these nodes (and with initial time in the span of the core) and at nodes chosen with other seeding strategies. The results indicate that seeds chosen in span-cores of the highest order have consistently a large spreading power, which tends to increase with the order of the span-core considered.

Overall, our findings confirm that maximal span-cores in temporal networks play an important role in propagation processes on these networks. These results should in particular stimulate the design of models of temporal networks with non-trivial span-core structures, in addition to the usually considered statistics of contact durations and inter-contact times.

Our work has several limitations worth discussing. First, we have limited our investigations to the schematic SIS and SIR models, while real diseases have most often a more complicate description with a larger number of possible states for each individual (such as latent, asymptomatic,...). However, despite their simplicity, SIR and SIS models capture the main phenomenology of spreading processes, and these paradigmatic processes therefore constitute the first natural testing ground for understanding whether a certain type or node or structure is relevant in spreading processes^4,8,10,11,16,18,20^. This should nevertheless be kept in mind, and future work should also concern more realistic models of spread, in particular when addressing more concrete applications. Second, we consider here that the whole temporal network is known “in advance”, and the thought experiments we perform by removing structures are not causal. As described in the introduction, such causal interventions have been discussed in other works^17,18,21^ and correspond to a different type of studies. Here, our goal was to assert the importance of a certain type of temporal network structures in the unfolding of spreading processes. The next steps towards the effective design of applied strategies would be to relate the span-cores to interpretable events or features in a context of interest and to use such knowledge to devise actionable strategies, as done for instance in several works dealing with strategies towards controlling outbreaks in schools^22,23,38^.

Further work includes thus more realistic design and investigation of strategies, in particular under the hypothesis that the temporal network is not fully known but for instance only its main statistical properties^24,36^. Moreover, it would be interesting to investigate the role of span-cores in other types of dynamical processes on networks, such as opinion formation, complex contagion or synchronization processes.

## Methods

### Span-core decomposition and maximal span-cores

In a recent work, Galimberti et al.^26^ proposed an extension of core decomposition to temporal networks, whereby cores are associated with their temporal spans, i.e., intervals of contiguous timestamps for which the coreness property (i.e., of minimal connectivity) holds. Such cohesive temporal structures are named span-cores.

Let us consider a temporal graph $G = (V,T,\tau )$, where *V* is a set of nodes, $T = [0, 1, \ldots , t_{max}]$ is a discrete time domain, and $\tau : V \times V \times T\rightarrow \{0,1\}$ is a function defining for each pair of vertices $u,v \in V$ and each timestamp $t \in T$ whether the edge (*u*, *v*) exists in *t*. We denote $E = \{(u,v,t) \mid \tau (u,v,t) = 1 \}$ the set of all temporal edges. Given a timestamp $t \in T$, $E_t = \{(u,v) \mid \tau (u,v,t) = 1 \}$ is the set of edges existing at time *t*. Given a subset of nodes $S \subseteq V$, let $E_{\Delta }[S]$ be the set of edges connecting the nodes of *S* that exist in *all* timestamps $t \in \Delta$. We then define the temporal degree of a node *u* within the subgraph $G_{\Delta }[S] = (S , E_{\Delta }[S])$ as $d_\Delta (S,u) = |\{v \in S \mid (u,v) \in E_\Delta [S] \}|$. In other words, the temporal degree of *u* is the number of other nodes to which *u* is linked in all the timestamps of $\Delta$, without interruption.

#### Definition 1

($(k,\Delta )$-core) The $(k,\Delta )$-core of a temporal graph $G = (V,T,\tau )$ is (when it exists) a maximal and non-empty set of nodes $\emptyset \ne C_{k,\Delta } \subseteq V$, such that $\forall u \in C_{k,\Delta } : d_\Delta (C_{k,\Delta },u) \ge k$, where $\Delta \sqsubseteq T$ is a temporal interval and $k \in {\mathbb {N}}^+$.

The interval $\Delta$ and the integer *k* are referred to as span and order of the span-core, respectively. As it is well known, the cores of a static graph define a hierarchy. On the other hand, span-cores are not all nested into each other. Nonetheless, they exhibit containment properties. In particular, we say that a $(k,\Delta )$-core is contained into another $(k^{'},\Delta ^{'})$-core if $k^{'} < k$ and $\Delta \sqsubseteq \Delta ^{'}$.

The number of span-cores is quadratic in |*T*|. This is definitely not desirable when human inspection is of interest. Therefore, it is useful to focus only on the most relevant ones. Thus, Galimberti et al.^26^ introduced the concept of maximal span-core.

#### Definition 2

(Maximal span-core) A span-core $C_{k,\Delta }$ of a temporal graph *G* is said *maximal* if there does not exist any other span-core $C_{k',\Delta '}$ of *G* such that $k \le k'$ and $\Delta \sqsubseteq \Delta '$.

A span-core is thus identified as maximal if it is not dominated by any other span-core in terms of both order and span. Clearly, maximal span-cores resemble the idea of innermost core, i.e., the core of highest order, in the core decomposition of a static graph. However, maximal span-cores are not unique. Instead, there is at most one maximal span-core for every temporal interval.

We also recall here some basic ideas of the efficient algorithm to compute all span-cores in a temporal network^26^. A naive approach would involve executing a static core decomposition routine independently for each temporal interval $\Delta$. A more efficient procedure exploits the containment property in both its dimensions, coreness and temporal intervals. Indeed, given a temporal graph *G*, and a temporal interval $\Delta = [t_s,t_e] \sqsubseteq T$, let $\Delta _+ = [\min \{t_s+1,t_e\}, t_e]$ and $\Delta _- = [t_s, \max \{t_e-1,t_s\}]$. It holds that

$$\begin{aligned} C_{1,\Delta } \ \subseteq \ (C_{1,\Delta _+} \cap C_{1,\Delta _-}) \ = \ \bigcap _{\Delta ' \sqsubseteq \Delta } C_{1, \Delta '} . \end{aligned}$$

The algorithm^26^ takes advantage of this simple property by processing temporal intervals of increasing size (starting from size one) and, for each interval $\Delta$ of width larger than one, the core decomposition is initiated from $(C_{1,\Delta _+} \cap C_{1,\Delta _-})$, the smallest intersection of cores containing $C_{1,\Delta }$. This expedient produces a speed-up of orders of magnitude in the obtention of all span-cores.

The problem of computing the maximal span-cores of a temporal graph can also be addressed by the simple approach of extracting all span-cores and then filtering out those which are not recognized as maximal. However, theoretical properties that relate the maximal span-cores to each other prove that it is not required to compute the overall temporal core decomposition but it is possible to extract only the maximal span-cores. Note that this is a challenging design principle, as it contrasts the idea that a core of order *k* is typically computed from the core of order $k-1$. Theoretical findings provide bounds on the order of a maximal span-core and suggest a top-down algorithm that processes temporal intervals starting from the larger ones, in opposition to the method used to extract the entire span-core decomposition. The procedure does not search the whole span-core space and it has been empirically shown to be markedly more efficient compared to the approach based on filtering out non-maximal span-cores^26^.

The algorithms are detailed in Ref.^26^ and the code is publicly available on https://github.com/egalimberti/span_cores

### Datasets

We consider 8 data sets describing human interactions with high spatial and temporal resolutions in a variety of contexts: schools (at different levels and in different countries), conferences, workplace (office building) and hospital. These data are publicly available thanks to two independent collaborations. SocioPatterns^30^ gathers longitudinal data on physical proximity and face-to-face contacts of individuals in different contexts using a sensing platform based on wearable badges equipped with radiofrequency identification devices (RFIDs). Contact data are collected with a temporal resolution of 20 s. Further data describing human proximity are provided by Toth et al.^31^, who deployed a platform composed of wireless ranging enabled nodes (WRENs) in several schools in the USA. Each WREN collects signals from other WRENs in proximity at intervals of approximately 20 s. Signal strength criteria are used to select pairs of individuals wearing these WRENs located at distance lower than or equal to 1 meter: this is the practical definition used to define a contact between individuals at each time. For each data set, we aggregate all interactions in successive time-windows (timestamps) of 300 s. We thus obtain from each data set a temporal network in which nodes represent individuals, and a temporal edge is drawn in a timestamp *t* between two nodes if the two corresponding individuals have been in contact in this time-window.

The specific data sets we use are the following. The first 6 are provided by the SocioPatterns^30^ collaboration, and the last 2 by Toth et al.^31^.

- The *Primary School* data set contains the contact events between 242 individuals (232 children and 10 teachers) in a primary school in Lyon, during 2 days in October 2009^39^.
- The *High School* data set gives the interactions between 327 students of nine classes within a high school in Marseille, during 5 days in December 2013^35^.
- The *Hospital* data set describes the face-to-face interactions of patients and health-care workers (HCWs) in a hospital ward in Lyon, France during 1 week in December 2010. The study included 46 HCWs and 29 patients^40^.
- The *Conference* data set was collected during the ACM Hypertext 2009 conference, which took place between June 6, 2009 and July 1, 2009 in Turin, Italy. The data cover a period of 2 days and a half^41^.
- The *SFHH conference* data set describes the face-to-face interactions of 405 participants to the 2009 SFHH conference in Nice, France (June 4–5, 2009) citeGenois:2018.
- The *Workplace* data set contains the temporal network of contacts between individuals recorded in an office building in France in 2015^24^.
- The *Elementary School* data set contains the contact data associated with the 476 students in the 21 classes of a suburban elementary school in Utah (USA) on January 31, 2013 and February 1, 2013^31^.
- The *Middle School* data set describes the proximity interactions occurred on November 28 and 29, 2012 in an urban public middle school in Utah (USA)^31^.

Some more details are given in Table 2.

**Table 2:** Properties of the data sets considered here and of the span-cores with largest order or duration.

Data set and reference	Number of nodes	Number of temporal edges	Total duration (in number of timestamps)	Core of largest order: (order, duration, number of nodes)	Core of max duration: (order, duration, number of nodes)
Primary school	242	55k	208	(7, 1, 10)	(1, 27, 2)
High school	327	47k	492	(6, 1, 7)	(1, 50, 2)
Elementary school	339	41k	152	(9, 1, 13)	(1, 49, 5)
Middle school	590	68k	168	(10, 1, 11)	(1, 23, 2)
SFHH	403	21k	247	(9, 1, 10)	(1, 83, 2)
ACM hypertext conference	113	7k	424	(7, 1, 9)	(1, 38, 2)
Workplace	217	21k	1k	(7, 1, 24)	(1, 97, 2)
Hospital	75	9218	627	(6,1,7)	(1,55,2)

### Spreading processes and impact evaluation

We consider the two paradigmatic spreading processes Susceptible-Infectious-Susceptible (SIS) and Susceptible-Infectious-Recovered SIR, with parameters $\beta$ and $\nu$, as described in the main text: $\beta$ is the probability per unit time that a susceptible node in contact with an infectious one becomes infectious, and $\nu$ is the probability per unit time that an infectious node recovers spontaneously (becoming again susceptible in the SIS case, and recovered in the SIR case). These spreading processes are considered on the temporal network data: contagion can occur only along the temporal edges.

In the spreading mitigation scenario, our aim is to compare the unfolding and impact of these processes, for each data set, on the original temporal network and on temporal networks modified according to several intervention strategies. To quantify the differences between processes in original and altered networks, we resort to two common measures of the epidemic risk and study how its value is modified by the intervention. We first consider the value of the epidemic threshold, i.e., the critical value of disease transmissibility $\beta$ above which the spread is able to reach a large fraction of the population. The analytical method developed by Valdano et al.^32^, based on the approximation of the process by a Markov chain, allows to express the epidemic threshold for both processes in terms of the spectral radius of a matrix that encodes both network structure and disease dynamics. We use the Python package publicly available at http://github.com/eugenio-valdano/threshold to compute the threshold as a function of the recovery parameter $\nu$ for the various data sets and the various intervention strategies, and measure the impact of each strategy through the relative change in the threshold value.

We moreover consider the final size of the epidemic, i.e., the fraction of nodes that have been reached by the process when it ends. At the end of the SIR process, no infectious nodes are left and the epidemic size is given by the fraction of nodes in the R state. Note that, as the SIR epidemic might not end within the finite span of the data, the temporal network is repeated if needed until the process ends. For the original data, for each strategy and for each set of parameters $(\beta ,\nu )$ we simulate $N_{sim} = 1000$ SIR processes. We then compute the epidemic size ratio for each strategy and parameter values, defined as the ratio between the average epidemic size for processes on the reduced temporal network obtained according to a strategy and the average epidemic size for processes on the original temporal network:

$$\begin{aligned} \rho _{strategy}(\beta ,\nu ) = \frac{\langle R_{final} \rangle _{strategy}}{\langle R_{final}\rangle _{original}} \ . \end{aligned}$$

In the spreading maximization scenario, the goal is to assess the performance of the maximal span-cores as a tool to identify the nodes that,when infected, lead to wide propagation. We consider numerical simulations of the SIR process and we adopt the final size of the epidemics as a performance metric for a seeding strategy. For each seed, we simulate $N_{sim} = 100$ SIR processes starting at different timestamps. Again, if required, the temporal domain of a temporal network is repeated until the process end. We then compute the epidemic size ratio, in which the numerator and denominator are given by the average epidemic sizes for processes seeded according to a strategy and for randomly seeded processes, respectively:

$$\begin{aligned} \rho _{strategy}(\beta ,\nu ) = \frac{\langle R_{final} \rangle _{strategy}}{\langle R_{final}\rangle _{random}} \ . \end{aligned}$$

Clearly, although in the spreading mitigation and maximization frameworks the epidemic size ratios are defined similarly, in the former smaller values are desiderable while in the latter larger values are to be preferred.

### Mitigation strategies

We describe here in detail the targeted intervention strategies aimed at mitigating the spread of an epidemic process unfolding in a host population described by a temporal network. These interventions consist in removing temporal edges in the network, and different strategies consider different ways of choosing the edges to be removed. The strategies we put forward target the maximal span-cores of the given temporal network $G = (V, T, \tau )$. One possibility is to choose an a priori number $n_{msc}$ of maximal span-cores and remove all the corresponding edges. This can be interpreted as temporary isolation: a node *u* belonging to one of the chosen maximal span-cores $C_{k,\Delta }$ is isolated over the time interval $\Delta$. As different cores have different sizes, fixing $n_{msc}$ would however lead to different fractions of removed temporal edges for the different strategies. We thus fix the fraction *f* of temporal edges to be removed and remove them starting with the maximal span-cores taken in a chosen order. We consider here $f=20\%$ and show in the supplementary material the results of using $f=10\%$.

As the maximal cores can be classified along two properties, their order and their durations, we consider in fact two separate strategies:

- The top-*k* maximal span-cores strategy (kM for short) removes temporal edges starting from the maximal span-cores with highest order, independently of their duration. We denote by $G_{kM}$ and $E_{kM}$ the temporal network and set of temporal edges remaining after the intervention, respectively.
- The top-$\Delta$ maximal span-cores strategy ($\Delta$M for short) removes temporal edges starting from the maximal span-cores with longest duration, independently of their order. We denote by $G_{\Delta M}$ and $E_{\Delta M}$ the temporal network and set of temporal edges remaining after the intervention, respectively.

We denote by $n_T$ the total number of temporal edges removed: $n_T = f |E|$ where |E| is the set of temporal edges in the temporal network considered. For each strategy, we precise that: (1) at given duration or order, the cores are ordered randomly; (2) if removing all the edges of a span-core would lead to removing more than $n_T$ edges, edges are removed at random from that core until reaching exactly $n_T$ removed edges.

Table 1 gives for each strategy the properties of the span-cores removed for the two data sets considered in the main text. These properties are given in the Supplementary material for the other data sets.

The $n_T$ temporal edges removed are not removed uniformly along the timeline of the temporal network, and we denote by $n_t^{s}$ the number of temporal edges removed at timestep *t* for strategy *s* ($s=kM$ or $s=\Delta M$). We evaluate the effectiveness of these intervention strategies by comparing their impact to the one of several baselines. Each baseline consists in removing the same number $n_T$ of temporal edges from the temporal network. The two simplest baselines consist in removing these edges at random:

- Randomly trimmed network (for short RT): the simplest benchmark is the intervention in which $n_T$ edges are randomly removed over the temporal domain.
- Randomly trimmed by timestamp network (for short RTT): this strategy uses the knowledge of the timestamps in which the targeted span-cores are active, by removing exactly $n_{t}^s$ edges randomly at each timestamp *t*. There are therefore two such strategies, kRTT that removes $n_{t}^{kM}$ random edges at time *t* and $\Delta$RTT that removes $n_{t}^{\Delta M}$ random edges at time *t*.

Note that the two RTT strategies exploit the temporal information provided by the temporal core decomposition while the RT one does not.

We consider as well more sophisticated baselines based on node centrality measures computed on the time-aggregated network. Indeed, it is known for static networks that nodes with large degree or static coreness play important roles in spreading processes. We also consider the weighted counterparts of degree and coreness, strength and weighted coreness^34^. We recall that in the time-aggregated network, the degree of a node *u* is equal to the number of distinct nodes with whom *u* has been in contact, and the weight of an edge (*u*, *v*) gives the number of temporal edges between *u* and *v* in the temporal network.

For each centrality measure, the strategy works as follows:

- we first sort the nodes in decreasing order of their centrality;
- we then consider the nodes one by one, starting by the most central ones, and removing all the temporal edges to which it belongs over the entire temporal domain, until the stopping criterion is met (i.e., until $n_{T}$ edges have been discarded). If discarding all the interactions of a node would mean exceeding $n_T$, the temporal interactions to be removed for this node are chosen at random.

We thus obtain the four following strategies:

- The highest static degree strategy (SD) strategy;
- The highest static coreness (SC) strategy;
- The highest static strength (weighted degree, SWD) strategy;
- The highest static s-coreness (weighted coreness, SWC) strategy.

Finally, we consider the strategy tPR, which is based on a generalization of PageRank to temporal network^29^. Here, a temporal PageRank value is assigned to each node *u* at each timestamp *t*: tPR(*u*, *t*). The pairs (*u*, *t*) are ranked according to these values and we remove their temporal edges using this ranking until $n_T$ temporal edges have been removed.

### Seeding strategies

In the spreading maximization scenario considered for the SIR model, we consider several seeding strategies, i.e., choice of the initial seed of the spread (the first node in state I) aimed at favouring the spread. The idea underlying the proposed procedure is that nodes that represent influential spreaders are likely to belong to the span-cores of highest order at the timestamp in which the spread begins. We consider a fraction $f = 5 \%$ of the nodes of a temporal network: The top-k maximal span-core seeding strategy (for short *kM*) requires to carry out the following steps:

- we rank the nodes according to the value of the highest order of the span-cores they belong to;
- in decreasing order, we take each node as seed of $N_{sim}=100$ SIR processes until the fraction *f* of the total amount of nodes has been considered. For a given node, the spread starts at a timestamp randomly sampled from the union of the spans of the highest order span-cores it belongs to. We then compute the average size of the epidemic, averaged over the $N_{sim}=100$ processes.

Strategies based on randomness are not properly defined in this case since random seeds are exploited in order to construct the performance metric. Instead, baseline strategies are based on the same static node centrality measures used in the spread mitigation scenario, namely degree (strategy SD), strength (strategy SWD), coreness (strategy SC) and weighted coreness (strategy SWC). For each centrality measure, the associated strategy is implemented as follows:

- we rank the nodes according to their centrality;
- in decreasing order of centrality, we take each node as a seed of $N_{sim}=100$ SIR processes until the fraction *f* of the total amount of nodes has been considered. In order for the comparison to be fair, the same sequence of initial timestamps considered in the seeding strategy based on the highest order maximal span-cores is considered.

## Supplementary Information

Supplementary Information.
