# Supplementary material for: Relevance of temporal cores for epidemic spread in temporal networks
Source: Sci Rep. 2020 Jul 27;10:12529. doi: 10.1038/s41598-020-69464-3 (PMC7385111; doi:10.1038/s41598-020-69464-3)
Supplement: Supplementary file 1 — Supplementary Information. [file 41598_2020_69464_MOESM1_ESM.pdf]

Supplementary Material

"Relevance of temporal cores for epidemic spread in temporal networks"

Martino Ciaperoni, Edoardo Galimberti, Francesco Bonchi, Ciro Cattuto, Francesco Gullo, and Alain Barrat

Supplementary Note 1. Aggregated statistics of maximal span-cores for original and reshuffled data sets

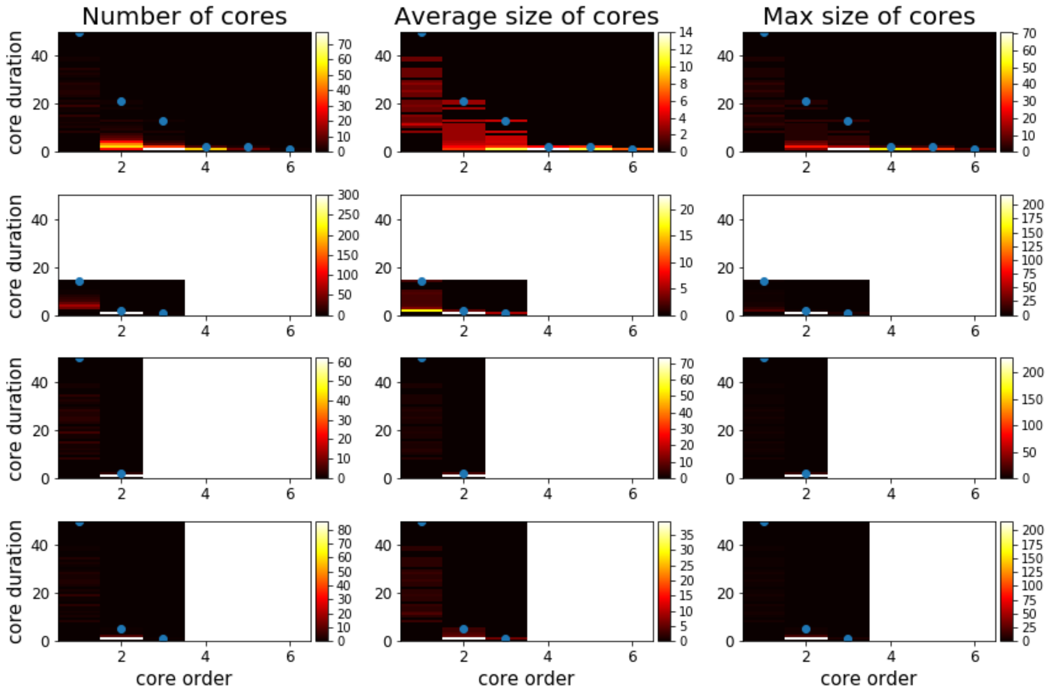

Figure S1. Aggregated statistics of the maximal span-cores for the original High School data set and for three reshuffled versions of this data set. Each panel shows with a color scale the property of cores with given core order (x-axis) and core duration (y-axis). Left plots: number of cores; Middle plots: average size of these cores; Right plots: size (number of nodes) of the largest of these cores. The blue dots give, for each order, the maximal duration of cores with that order. First row: original data. Second row: reshuffling R1, noted $P[w,t]$ in[1], which shuffles the timestamps among the temporal edges, thus preserving aggregated network and its weights and the global activity timeline. Third row: reshuffling R2, noted $P[\mathscr{L}, p(t,\tau)]$ in[1], which shuffles the contacts while keeping their starting time and duration, thus preserving contact duration statistics, global activity timeline and structure of the aggregated network. Fourth row: reshuffling R3, noted $P(k, p_{\mathscr{L}}(\Theta))$ in[1], for which one shuffles the links of the aggregated network according to the procedure of Sneppen & Maslov[2]: this preserves the activity timeline and statistics of contact durations but reshuffles any static structure.

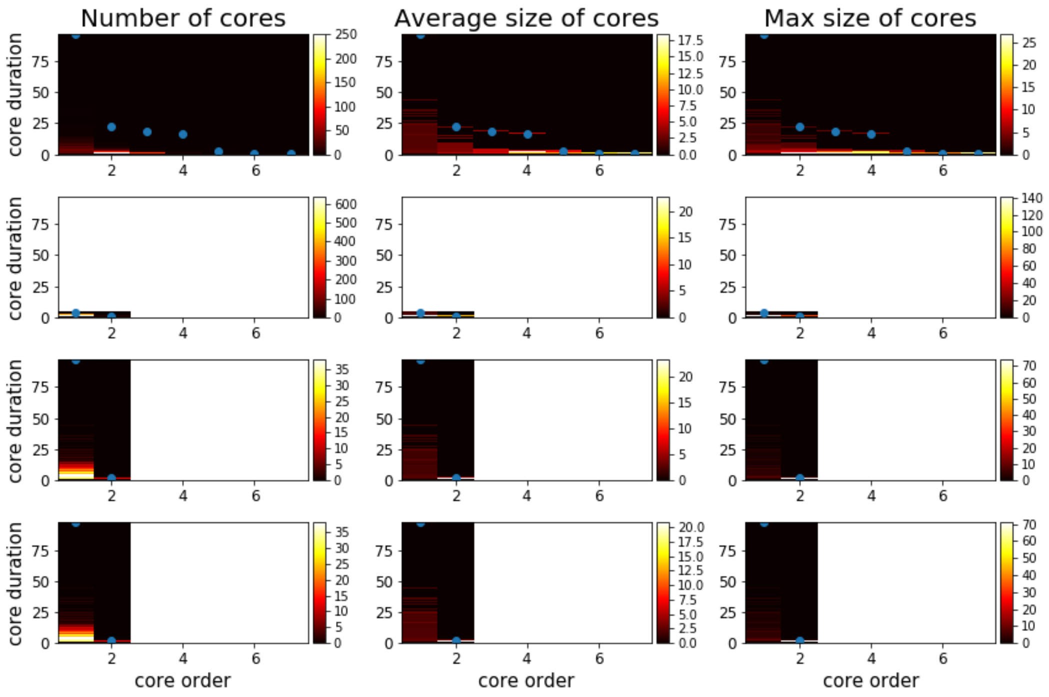

Figure S2. Same as Figure S1, for the Workplace data set.

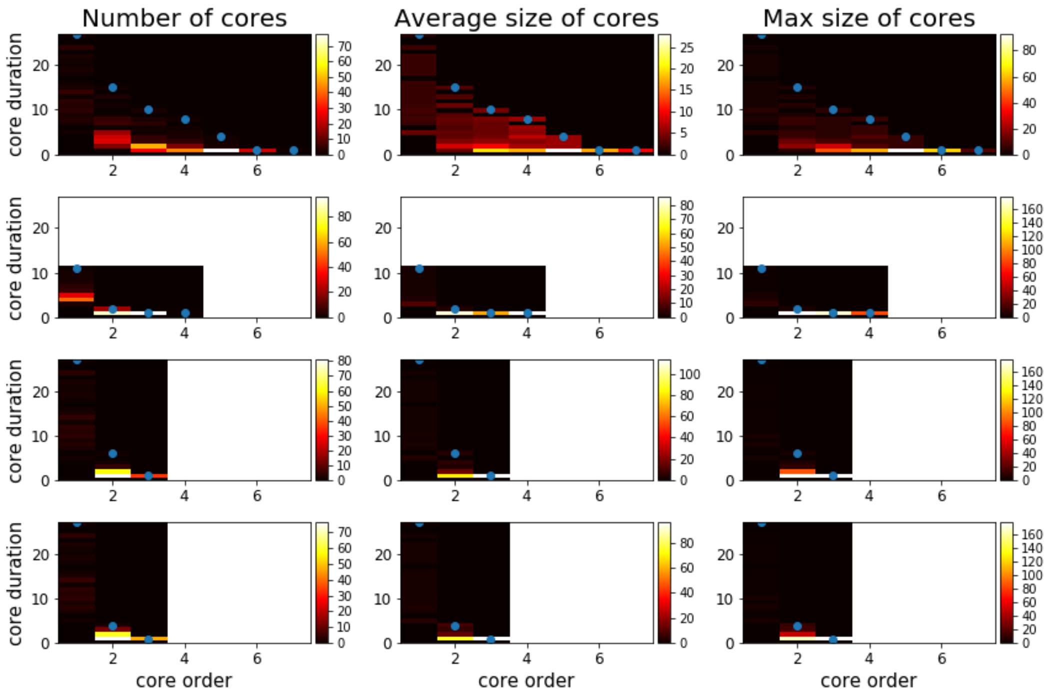

Figure S3. Same as Figure S1, for the primary school data set.

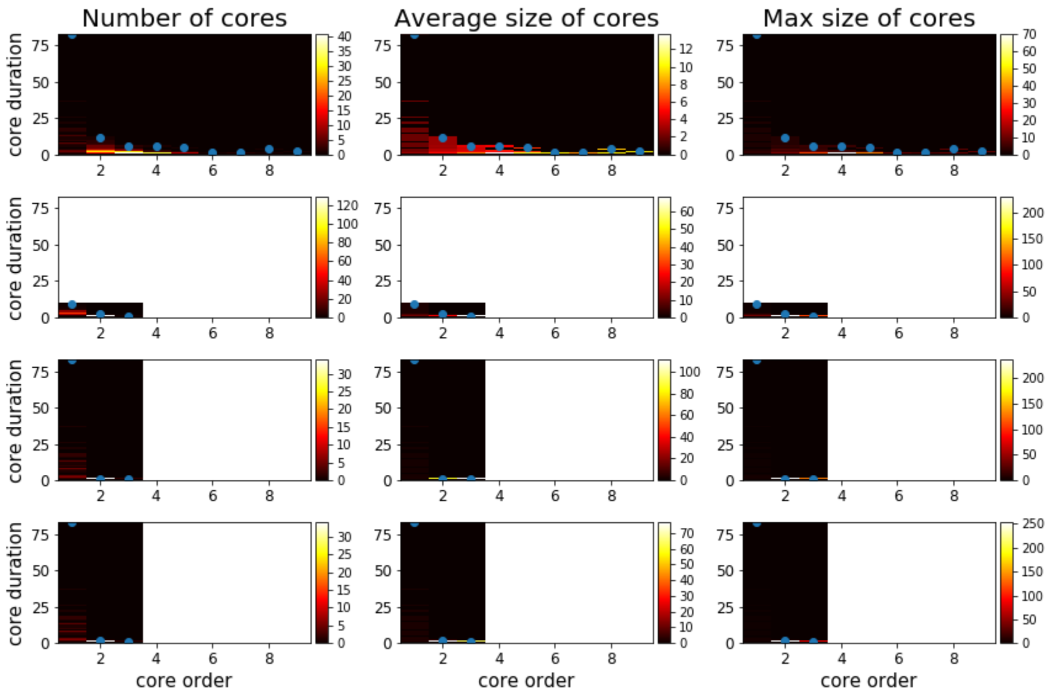

Figure S4. Same as Figure S1, for the SFHH data set.

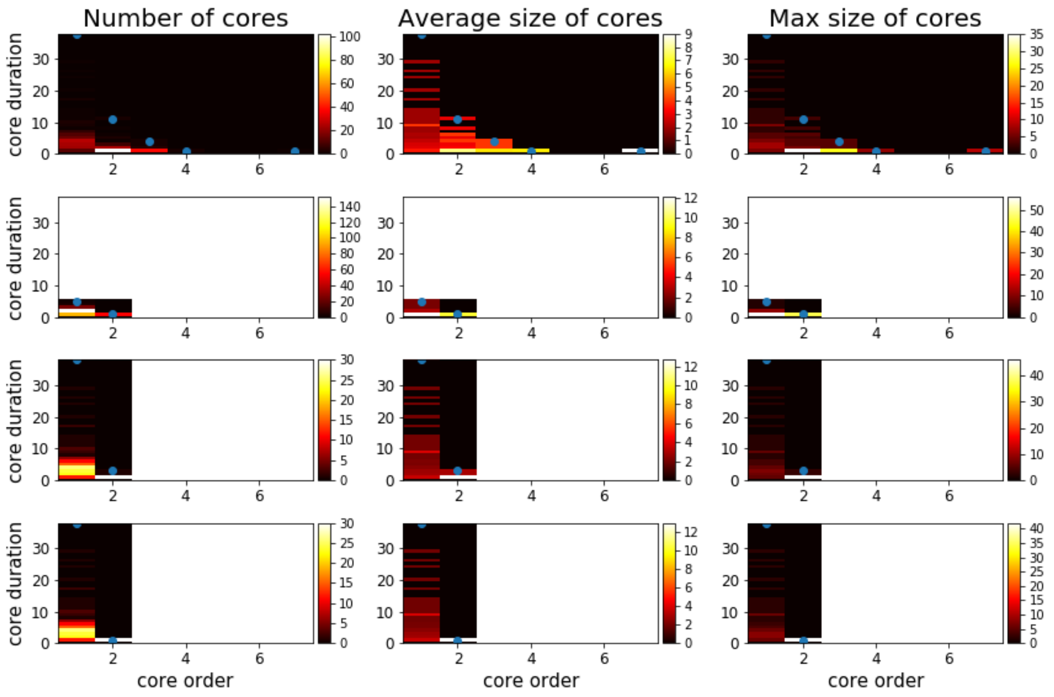

Figure S5. Same as Figure S1, for the ACM Hypertext data set.

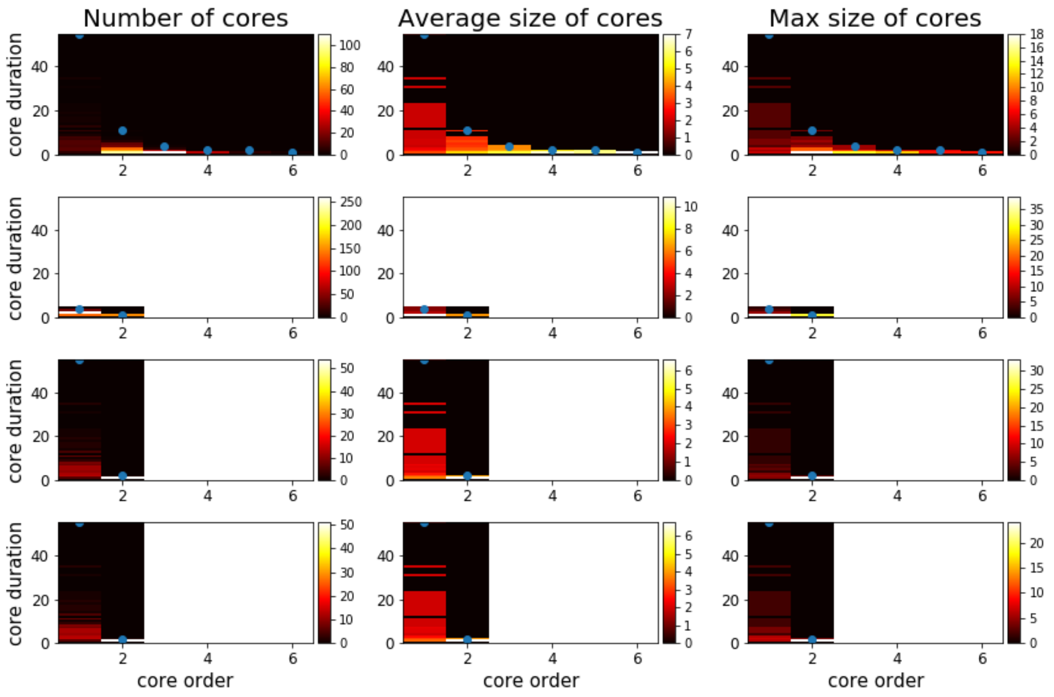

Figure S6. Same as Figure S1, for the Hospital data set.

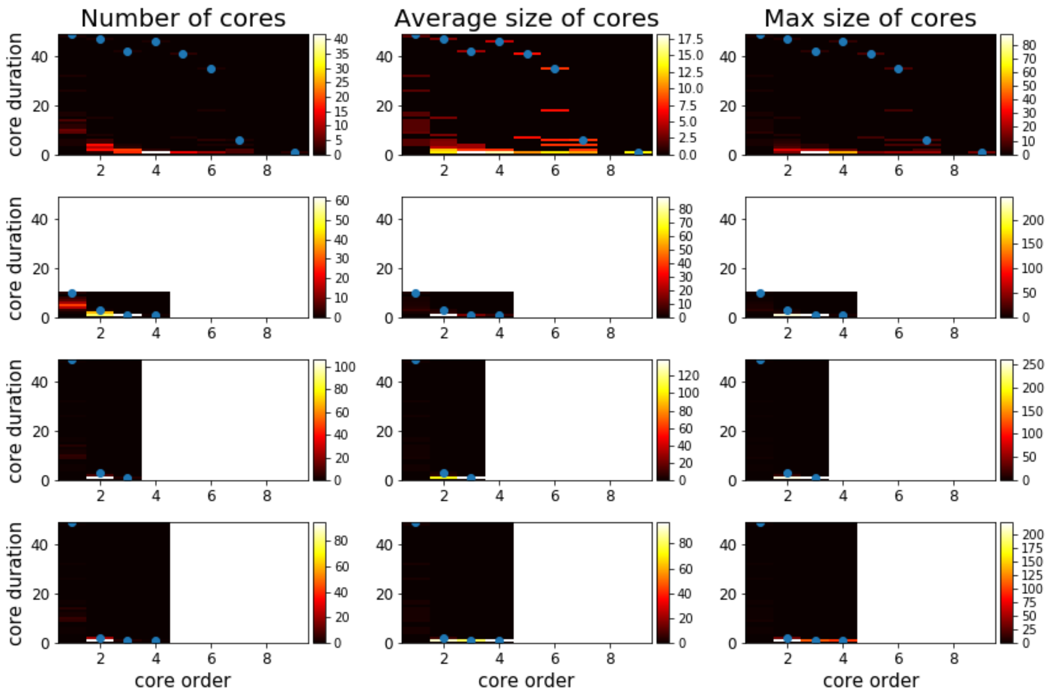

Figure S7. Same as Figure S1, for the Elementary School data set.

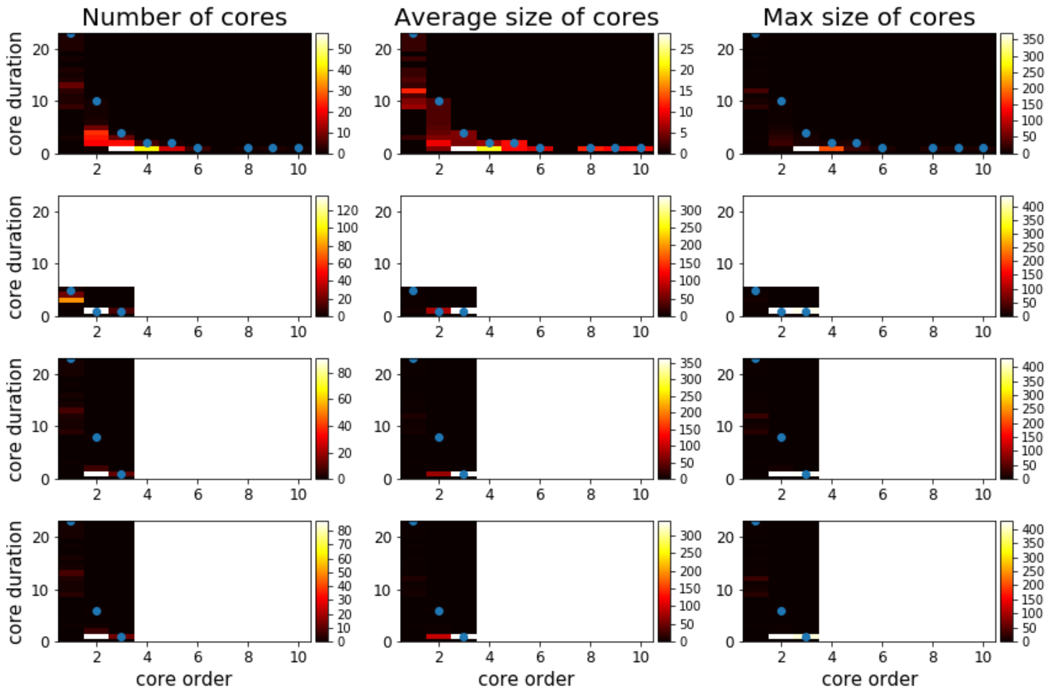

Figure S8. Same as Figure S1, for the Middle School data set.

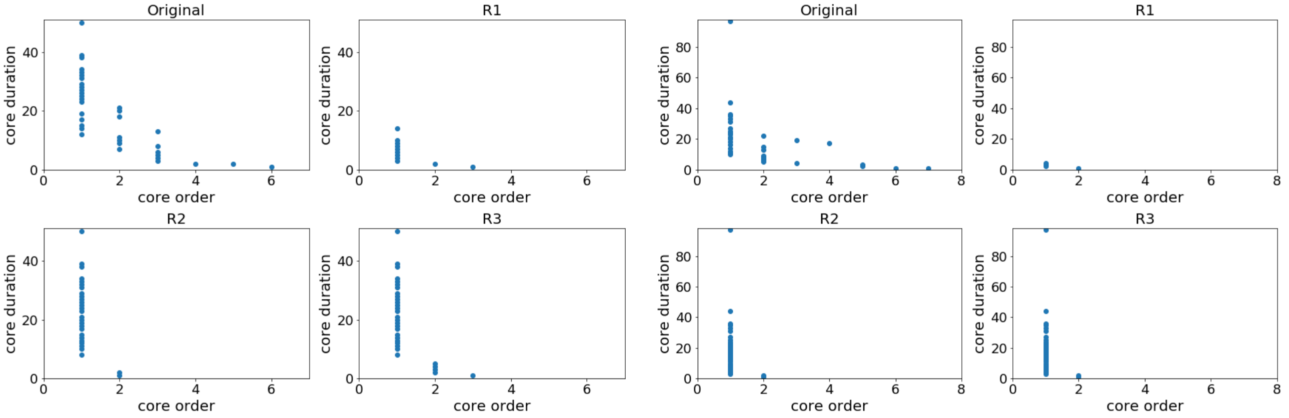

Figure S9. Maximal values of the order and duration of the span-cores of the High school data set (left) and of the Workplace data set (right). Each blue dot gives either the largest observed duration of maximal span-cores of a given order, or the largest observed order of maximal span-cores of a given duration, for the data set Primary School and for three reshuffled versions of the data set (See Fig. S1 for the definition of the reshuffling procedures R1, R2, R3.)

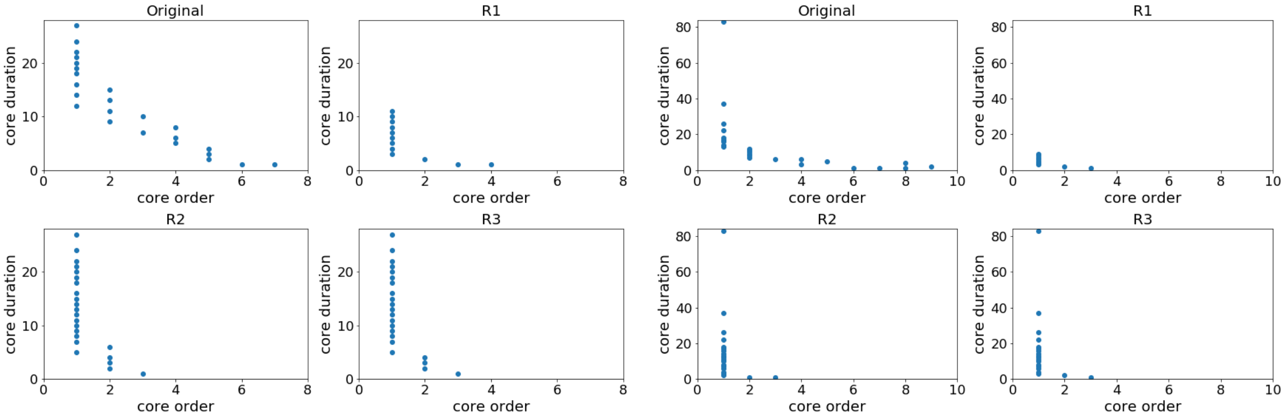

Figure S10. Same as Figure S9, for the Primary School data set (left) and the SFHH data set (right).

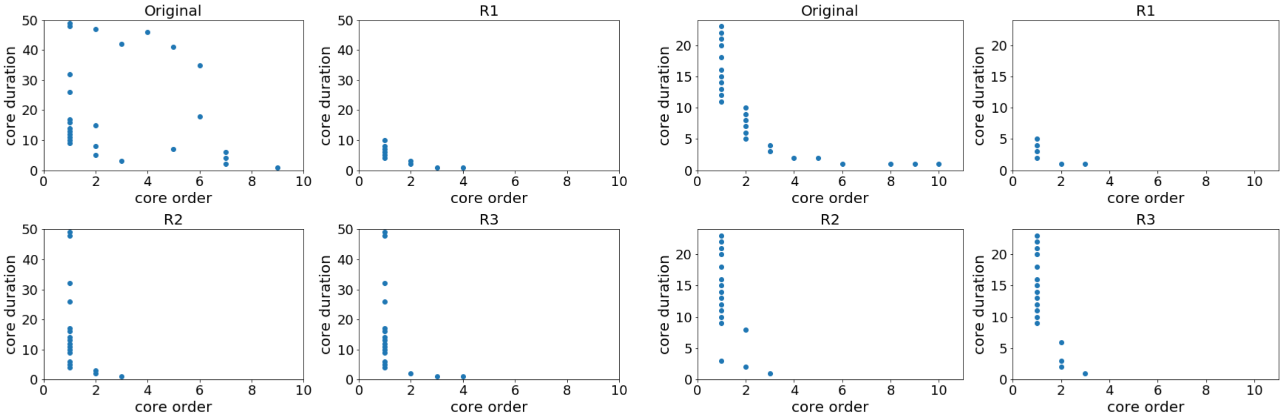

Figure S11. Same as Figure S9, for the Elementary School data set (left) and the Middle School data set (right).

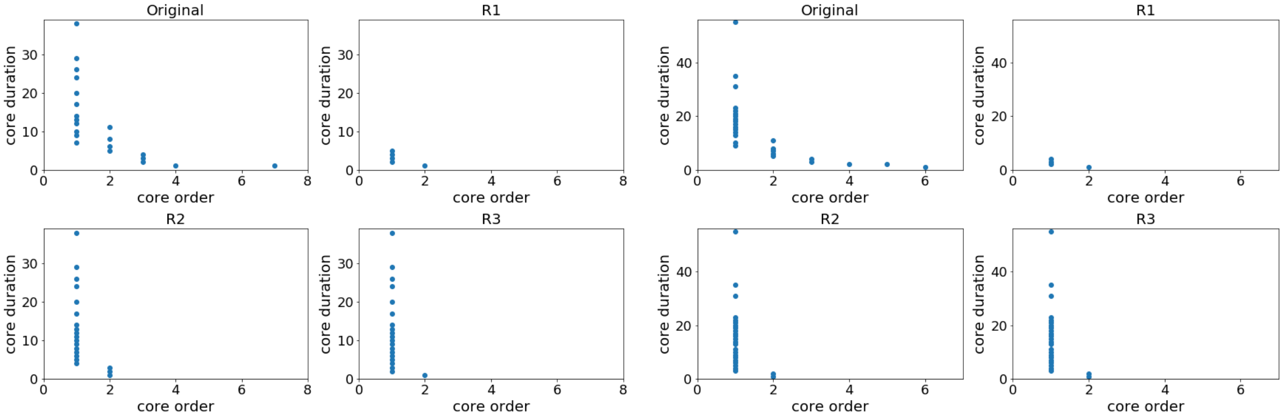

Figure S12. Same as Figure S9, for the ACM Hypertext data set (left) and the Hospital data set (right).

Supplementary Note 2. Static vs. dynamic coreness based centrality measures

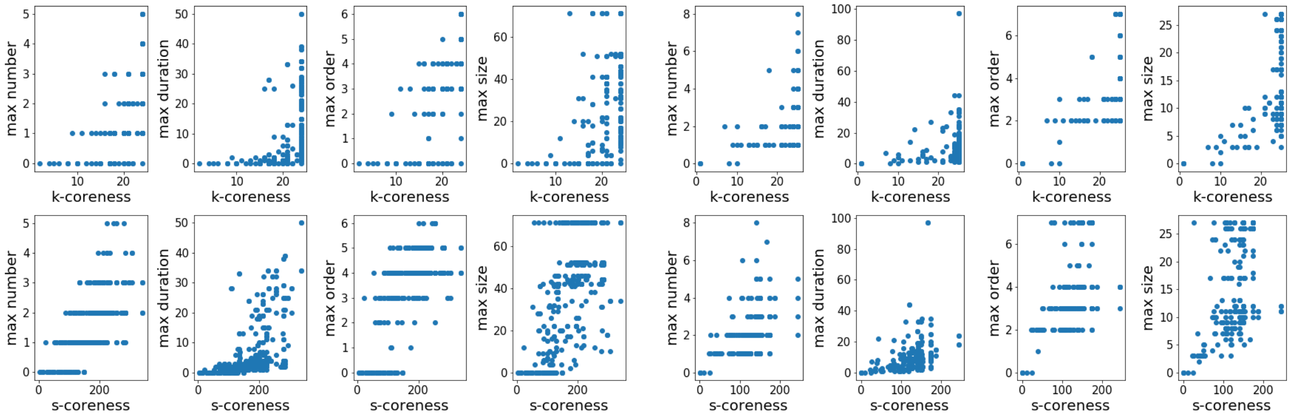

Figure S13. Scatterplot of node metrics related to the span-cores it belongs to vs. static metrics, for the High School data set (left) and the Workplace data set (right). The static metrics are given for the top and bottom row by respectively unweighted and weighted coreness. Each blue dot represents a node. The span-cores-related metrics are obtained, for each node, by following over time the number, durations, orders and sizes of the maximal span-cores it belongs to at each time step, and taking the maximal encountered value of these quantities in the whole timeline.

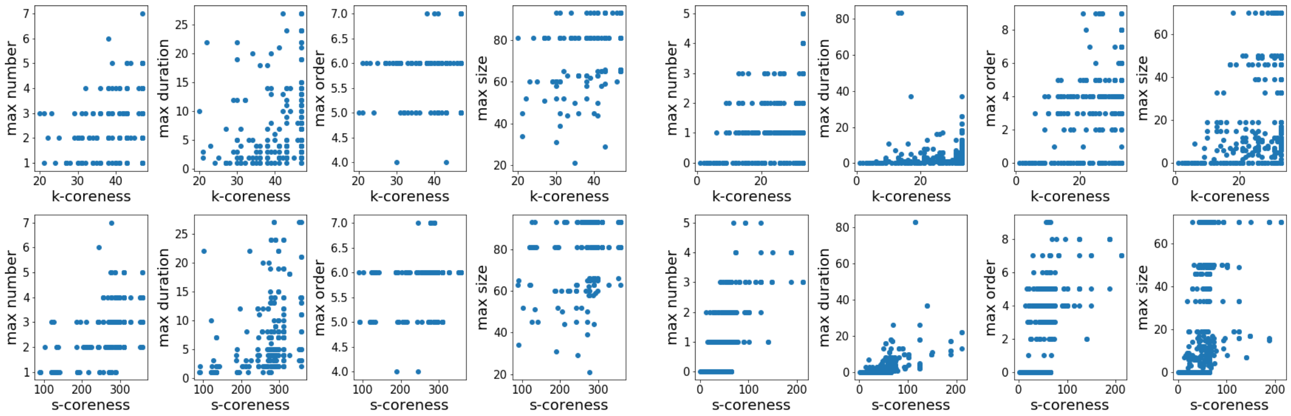

Figure S14. Same as Figure S13, for the Primary School data set (left) and the SFHH data set (right).

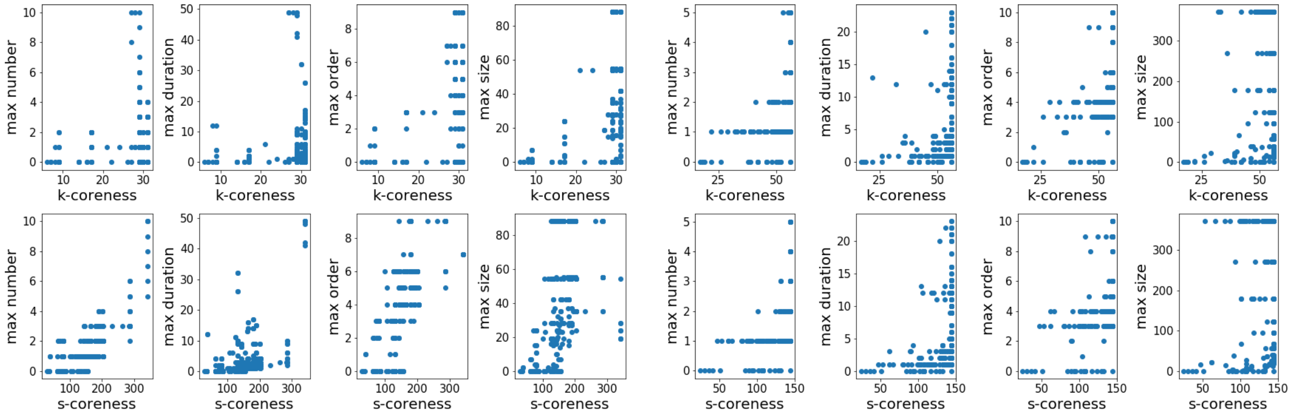

Figure S15. Same as Figure S13, for the Elementary School data set (left) and the Middle School data set (right).

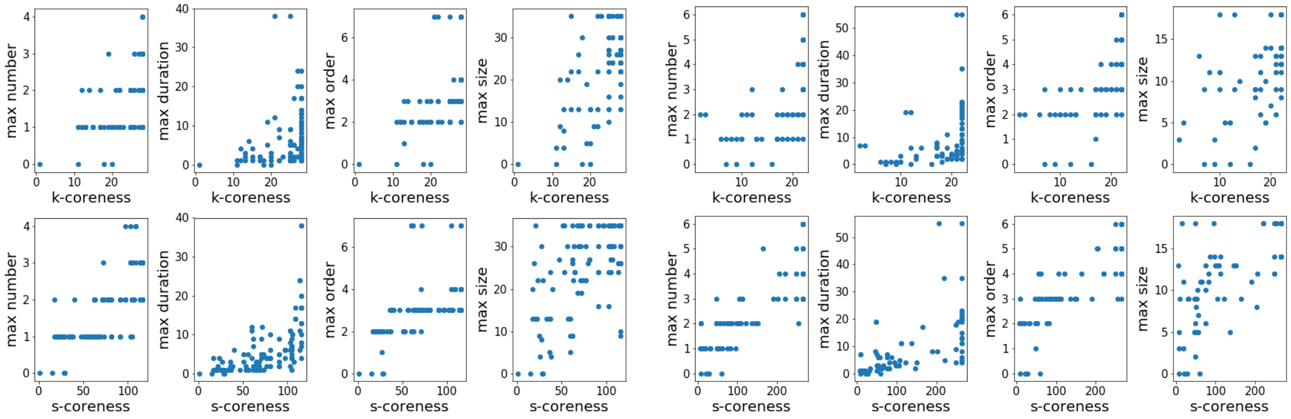

Figure S16. Same as Figure S13, for the ACM Hypertext data set (left) and the Hospital data set (right).

Supplementary Note 3. Properties of the (maximal) span-cores

Dataset and reference	number of span-cores	number of maximal span-cores	average size	average duration	average order
Primary school	4715	409	12.74	3.67	3.31
High school	12514	456	6.73	4.91	2.53
Middle school	3024	281	13.12	3.47	2.88
Elementary School	4334	212	9.83	5	3.29
SFHH Conference	6636	283	5.86	3.66	2.71
ACM Hypertext	3881	326	4.84	3.03	1.76
Workplace	16496	788	3.85	3.74	1.84
Hospital	8218	568	4.1	3.16	2.20

Table S1. Information concerning the temporal core decomposition of the datasets. The last three columns (average size, average duration and average order) are referred to the maximal span-cores only.

Supplementary Note 4. Properties of the targeted maximal-span cores

Data set	strategy	n_{msc}	$\langle k \rangle$	$\langle \lvert\Delta\rvert \rangle$	$\langle n \rangle$	$\langle e \rangle$
Primary School	largest order cores	100	5.25	1.07	21.71	112.46
Primary School	largest duration cores	167	2.18	6.15	3.86	92.48
Middle School	largest order cores	241	3.17	2.07	14.29	66.61
Middle School	largest duration cores	275	2.89	3.64	10.29	62.05
Elementary School	largest order cores	181	3.66	3.51	11.022	99.46
Elementary School	largest duration cores	191	3.32	5.45	8.76	104.95
ACM HT Conference	largest order cores	110	2.5	1.92	4.44	17.07
ACM HT Conference	largest duration cores	70	1.17	8.10	2.32	26.32
SFHH Conference	largest order cores	90	4.05	1.92	8.92	99.46
SFHH Conference	largest duration cores	127	2.25	6.53	3.43	104.95
Hospital	largest order cores	101	3.49	1.42	5.07	23.61
Hospital	largest duration cores	15	1	24.26	2	127.40

Table S2. Basic properties of the maximal span-cores removed in each of the targeted strategies ($f = 20\%$). n_{msc}: number of maximal span-cores with all temporal edges removed; $\langle k \rangle$: average order of the targeted maximal span-cores; $\langle \lvert\Delta\rvert \rangle$: average duration of the targeted maximal span-cores; $\langle n \rangle$: average number of nodes in the targeted maximal span-cores; $\langle e \rangle$: average number of temporal edges removed per time step impacted by the strategy.

Supplementary Note 5. SIS results for all data sets

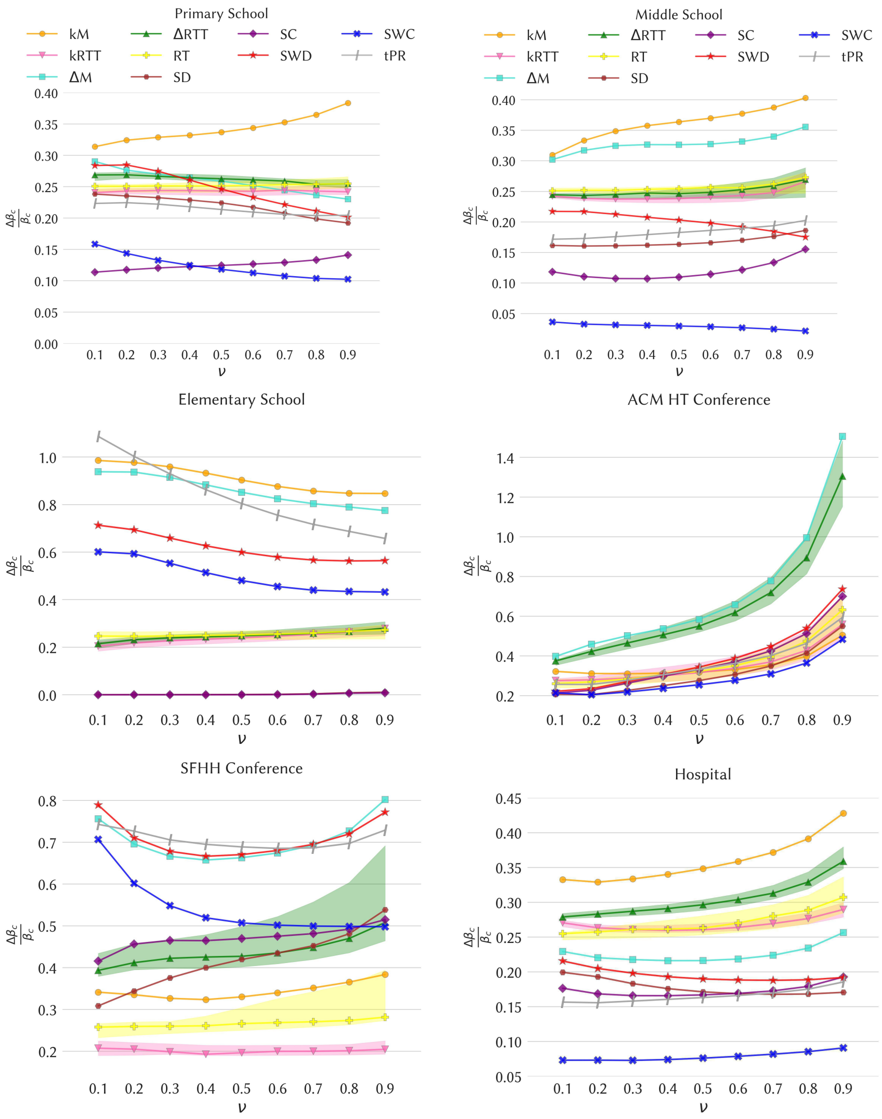

Figure S17. Impact of the intervention strategies as measured by the change in the epidemic threshold of SIS processes ($f = 20\%$). In each panel we plot for the various strategies the relative change $\Delta\beta_c/\beta_c$ in the epidemic threshold as a function of the recovery rate ν. For each strategy based on random choices, we show the confidence interval (computed using 30 samples) between the 5th and 95th percentiles as shaded areas.

Supplementary Note 6. SIS results for $f = 10\%$

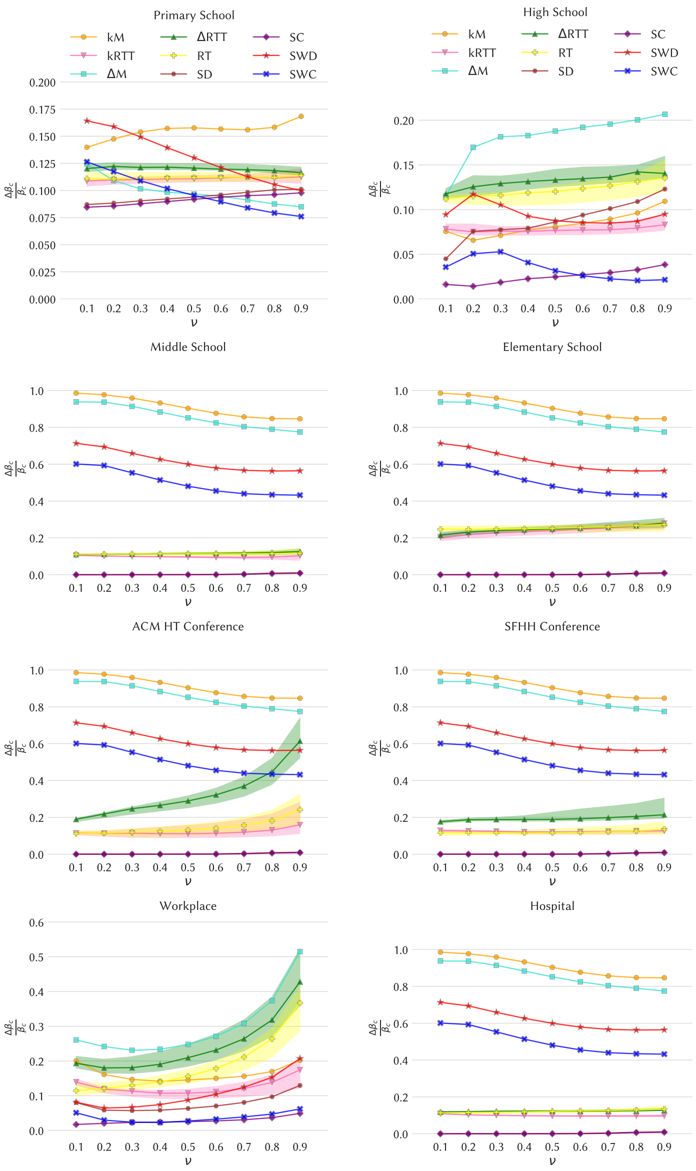

Figure S18. Impact of the various intervention strategies as measured by the relative change $\Delta\beta_c/\beta_c$ in the epidemic threshold of SIS processes, for a fraction $f = 10\%$ of temporal edges removed. For each strategy based on random choices, we show the confidence interval (computed using 30 samples) between the 5th and 95th percentiles as shaded areas.

Supplementary Note 7. Spread mitigation results for the SIR process

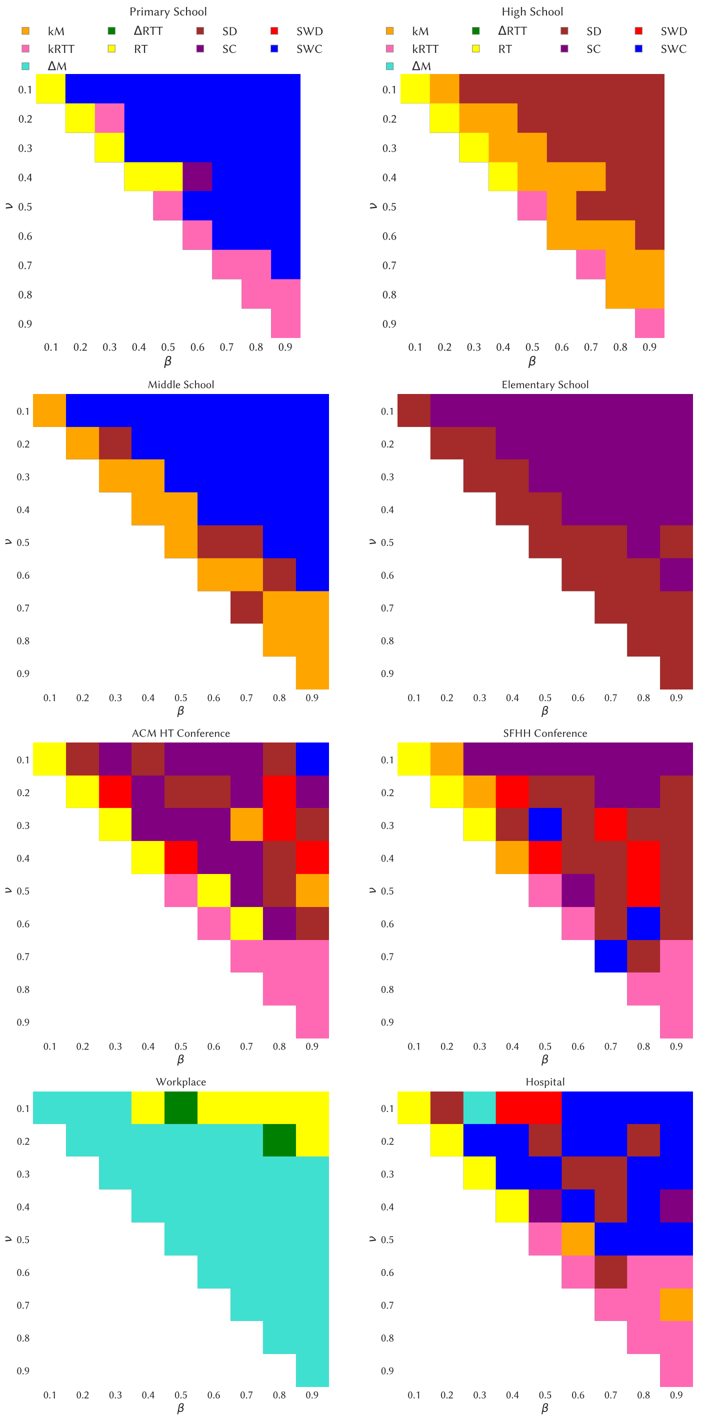

Figure S19. Heatmap indicating the intervention strategy leading to the lowest epidemic sizes, for each combination of spreading parameter values. Here $f = 20\%$.

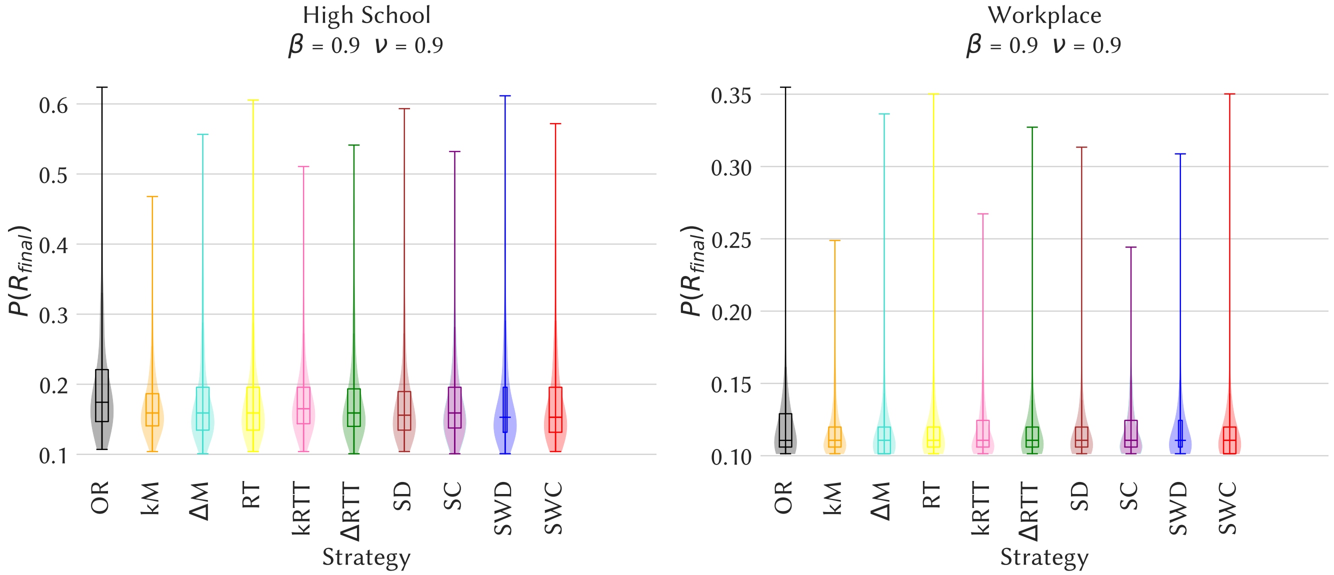

Figure S20. Distributions of final epidemic sizes for some illustrative cases, for an SIR process on the original temporal network (OR) and for the various mitigation strategies ($f = 20\%$). Left column: High School. Right column: Workplace.

Supplementary Note 8. Results of the seeding strategies for the SIR process, for all data sets (spread maximization)

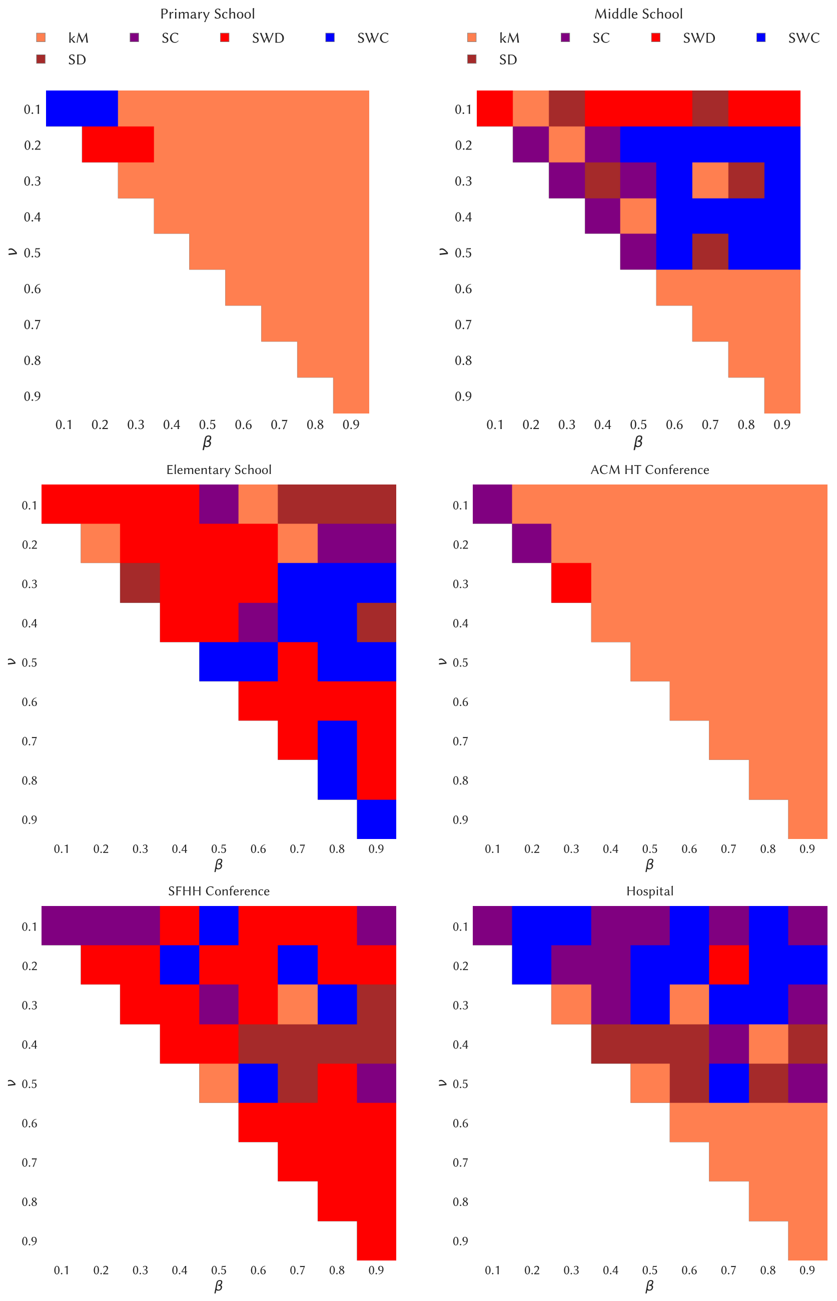

Figure S21. Heatmap indicating the intervention strategy leading to the largest ratio of final sizes, for each combination of spreading parameter values. For the Elementary School and SFHH Conference data sets, no seeding strategy is consistently optimal, and we show in Figure S22 that the distributions of the final epidemic sizes are in fact quite similar for the various strategies.

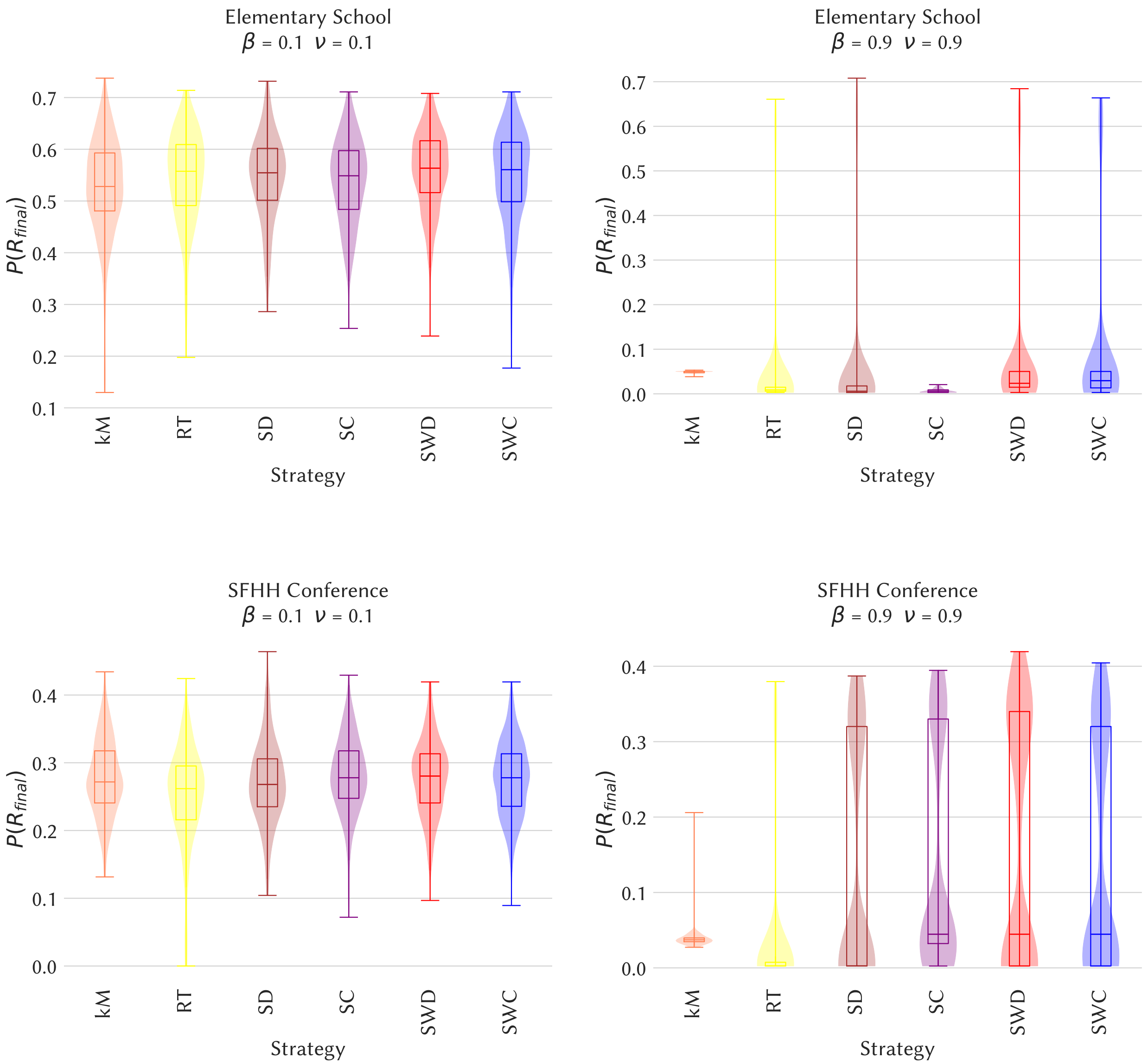

Figure S22. Distributions of final epidemic sizes in the Elementary School and SFHH Conference data sets for two illustrative cases of parameter values, for the various seeding strategies considered for the SIR process.

Supplementary Note 9. Effect of the order of the maximal span-cores in the SIR seeding

	minimum order		average order		maximum order	
Data set	$(n,\|\Delta\|, k)$	$\frac{\langle R_{final}\rangle}{\langle R_{final}\rangle_{random}}$	$(n,\|\Delta\|, k)$	$\frac{\langle R_{final}\rangle}{\langle R_{final}\rangle_{random}}$	$(n,\|\Delta\|, k)$	$\frac{\langle R_{final}\rangle}{\langle R_{final}\rangle_{random}}$
Primary school	(2,14,1)	1.39	(10,1,3)	5.08	(10, 1, 7)	33.35
High school	(4,15,1)	1.01	(7,1,3)	1.1	(7, 1, 6)	4.82
Middle school	(8,10,1)	2.23	(11,1,3)	4.12	(11,1,10)	5.17
Elementary school	(4,9,1)	3.16	(13,1,3)	4.82	(13,1,9)	4.51
ACM HT Conference	(9,1,1)	1.44	(9,1,2)	1.97	(9,1,7)	2.76
SFHH Conference	(4,1,1)	1.62	(10,2,3)	5.56	(10,2,9)	5.03
Workplace	(4,1,1)	1.38	(8,1,2)	1.74	(8,1,7)	3.46
Hospital	(4,1,1)	1.33	(7,2,2)	2.39	(7,1,6)	3.52

Table S3. Some properties (number of nodes n, duration $|\Delta|$, order k) of a maximal span-core of minimum, average and maximum order, and associated epidemic size ratio value $\frac{\langle R_{final}\rangle}{\langle R_{final}\rangle_{random}}$.

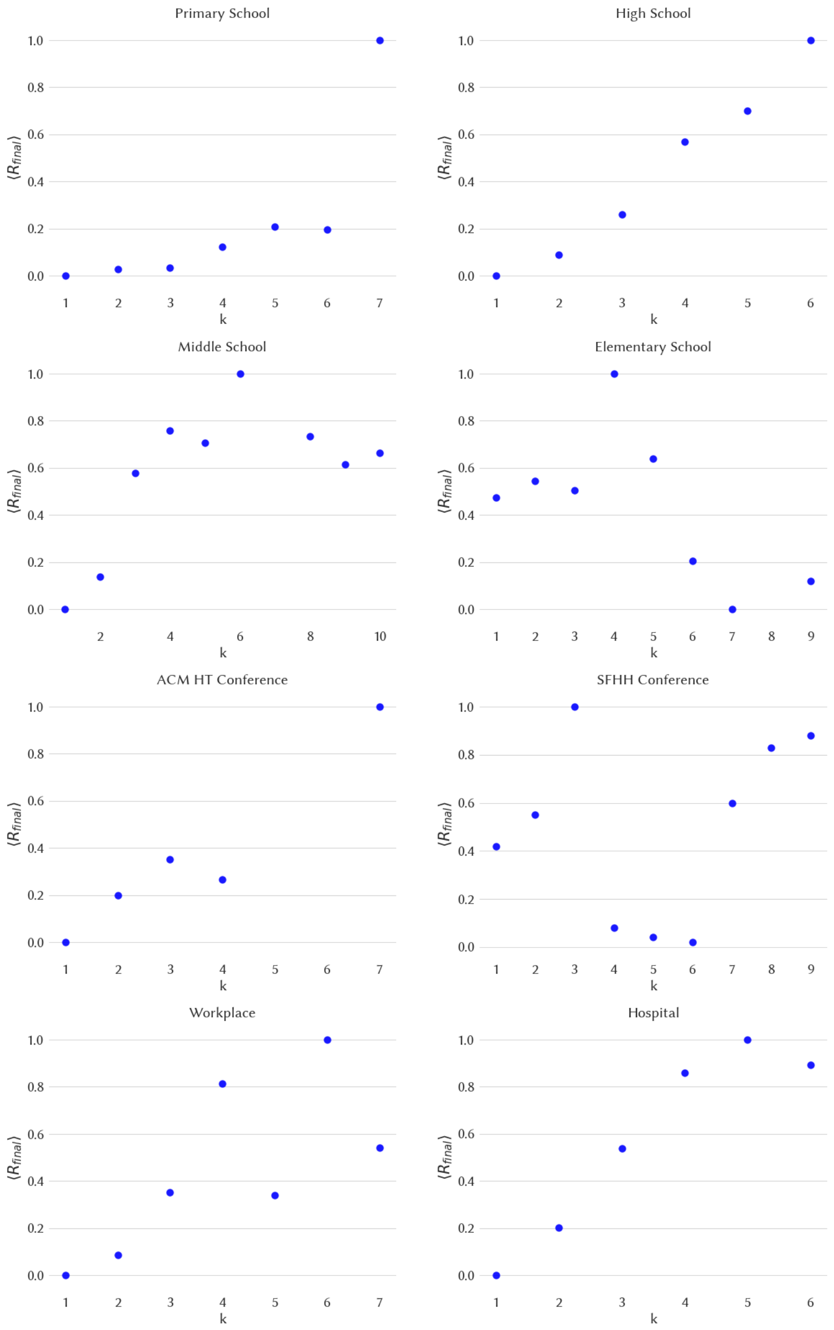

Figure S23. Effect of the order of the maximal span-cores in SIR processes for $\beta = 0.9$ and $\nu = 0.9$. In each panel we plot the average epidemic final size as a function of the order k of the maximal span-core to which the seed belongs.

References

1. Gauvin, L. *et al.* Randomized reference models for temporal networks. *arXiv* arXiv:1806.04032 (2018).

2. Maslov, S. & Sneppen, K. Specificity and stability in topology of protein networks. *SCIENCE* **296**, 910–913, DOI: 10.1126/science.1065103 (2002). http://www.sciencemag.org/content/296/5569/910.full.pdf.
